# Calcium‐dependent cooperativity and stability of Titin's tandem I82‐I83 domains

**DOI:** 10.1002/pro.70378

**Published:** 2025-11-12

**Authors:** Colleen M. Kelly, Janette Jerusal, Mark Pfuhl, Matthew J. Gage

**Affiliations:** ^1^ Department of Chemistry University of Massachusetts Lowell Lowell Massachusetts USA; ^2^ UMass Movement Center University of Massachusetts Lowell Lowell Massachusetts USA; ^3^ School of Cardiovascular Medicine and Sciences and Randall Centre, King's College London, Guy's Campus London UK

**Keywords:** calcium‐binding, N2A region, tandem immunoglobulin domains, titin

## Abstract

The muscle protein titin spans half a sarcomere, from M‐line to Z‐disk, and is essential for both active and passive stretch. The N2A region of titin plays a critical role in various regulatory processes through its binding interactions. Located at the C‐terminus of the N2A region, adjacent to the PEVK region, are the I82 and I83 domains, which are key to binding calpain/p94. However, this interaction is absent in the *mdm*‐mouse model, which contains an 83‐amino acid deletion spanning the C‐terminus of the I83 domain and the N‐terminus of the PEVK region, leading to muscular dystrophy with myositis. This *mdm*‐deletion disrupts the structure of the I83 domain, preventing normal force enhancement in the presence of calcium and inhibiting eccentric contractions. Our lab has demonstrated that the I83 domain exhibits calcium sensitivity at concentrations similar to those found in active muscle. In this current study, we further demonstrate that the tandem I82‐I83 domains exhibit cooperative unfolding, as seen by a single unfolding event, and that calcium enhances the stability of the tandem I82‐I83 domains. The NMR structure of this construct exhibits a tighter interface between I82 and I83 than is observed in the crystal structure, suggesting that the two structures might represent the structure in the relaxed state versus the structure under force. The calcium response of these domains is hypothesized to affect the function of the N2A region during muscle activation.

## 1 INTRODUCTION

The muscle protein titin plays significant roles in passive tension, sarcomere organization and as has been appreciated more recently, force in active muscle (Dutta et al., 2018; LeWinter et al., 2007; Linke et al., 1999; Maruyama, 1997; Powers et al., 2014; Rivas‐Pardo et al., 2020; Wang et al., 1979; Webb, 2003). While the sliding filament model can effectively explain concentric contractions in active muscle (Huxley, 1957), titin is believed to play a key role in the force generated during eccentric or lengthening contractions (Herzog, 2014a; Herzog, 2018; Powers et al., 2016; Schappacher‐Tilp et al., 2015). Early research on titin revealed a Ca^2+^‐dependent role for titin in proper muscle function (Kellermayer & Granzier, 1996; Leonard & Herzog, 2010; Powers et al., 2014; Schappacher‐Tilp et al., 2015). In skeletal muscle, and the N2BA isoform of cardiac muscle, a unique N2A region is located between the proximal Ig repeats and the PEVK region (Trombitas et al., 2001). This region plays a critical role in a variety of important regulatory and signaling interactions in the muscle (Belgrano et al., 2011; Hayashi et al., 2008; Miller et al., 2003).

**FIGURE 1:**
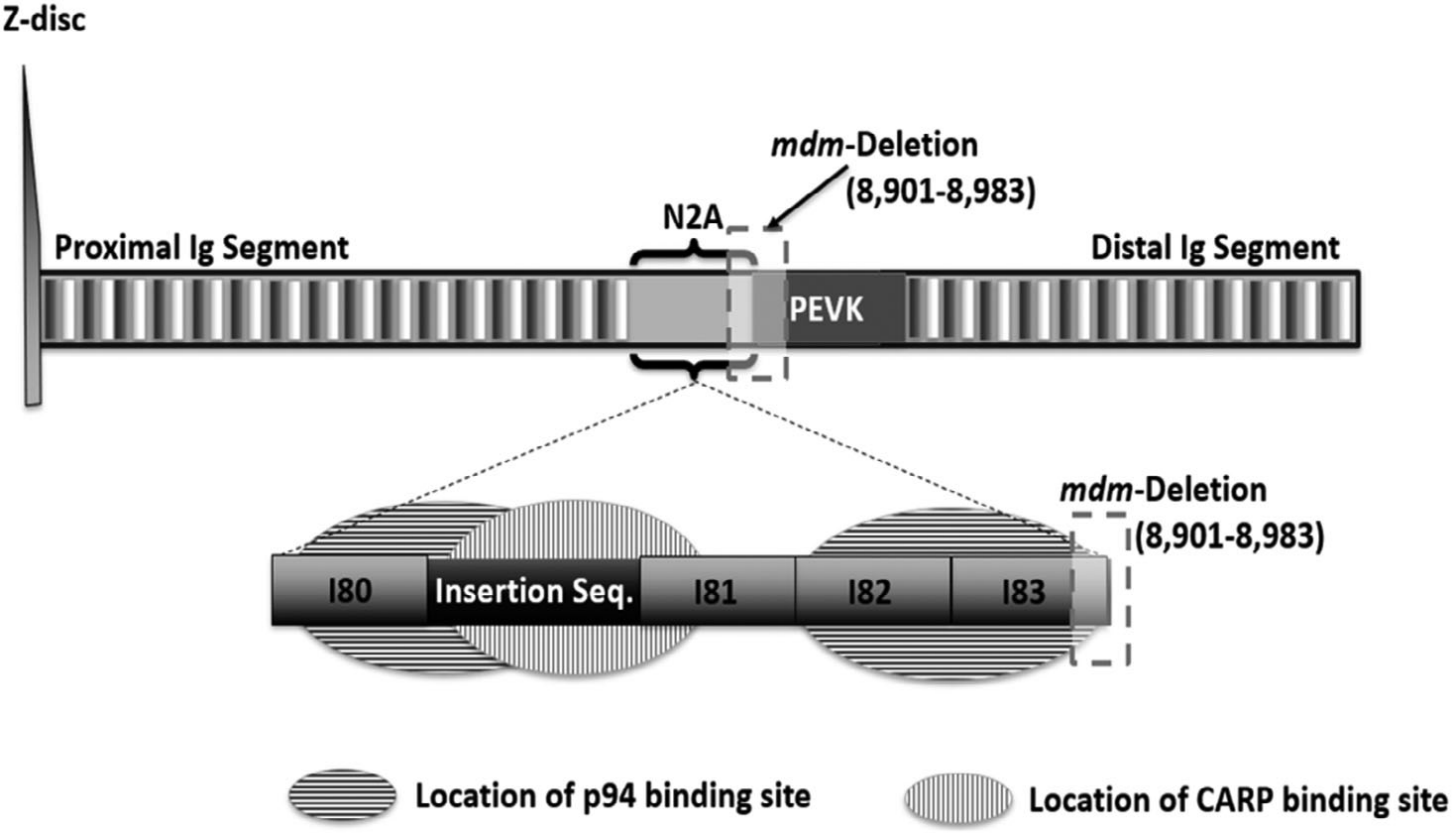
Calpain and ankyrin repeat protein binding sites and *mdm*‐deletion locations within the N2A region of titin. The I‐band of titin is shown with the N2A region of titin identified between the proximal Ig segment and the PEVK region. Unique features of the tandem Ig domains in the N2A region are highlighted including two calpain‐3 binding sites, which are identified by horizontal shading. Calpain‐3 (also called p94) is a muscle‐specific calpain. One site spans the I82 and I83 domains while the other spans I80 and the insertion sequence. A cardiac ankyrin repeat protein (CARP) binding site is identified by vertical shading at the insertion sequence, abutting the I81 domain. The *mdm*‐deletion truncates the I83 domain and the PEVK region and is crucial for proper muscle function (Miller et al., 2003; Ono et al., 2016).

Several models suggest that there is a calcium‐dependent interaction between actin and the N2A region of skeletal titin and the N2BA isoform of cardiac titin (Herzog, 2014a; Herzog, 2014b; Nishikawa et al., 2012; Nishikawa, Dutta, et al., 2020). Recent studies have supported these models, showing that the N2A region binds to actin, with this interaction being enhanced in the presence of calcium (Dutta et al., 2018). The N2A region contains four immunoglobulin (Ig) domains (I80 – I83), with a unique helical insertion between domains I80 and I81 (Figure 1) (Bang et al., 2001; Labeit & Kolmerer, 1995; Tiffany et al., 2017). This region acts as a signaling hub where the cardiac ankyrin repeat protein (CARP) binds to the UN2A‐I81 domains in N2A and calpain‐3/p94 is thought to bind to the I80‐UN2A and I82‐I83 domains (reviewed in (Adewale & Ahn, 2021; Hessel & Linke, 2021; Nishikawa, Lindstedt, et al., 2020)). A recent review discussing the calcium‐dependent binding between the N2A region and actin, suggested that the I83 domain may play a pivotal role, either as part of the actin‐binding site or through a calcium‐binding event that enhances the N2A‐actin interaction (Nishikawa, Dutta, et al., 2020). While several binding partners have been identified for the N2A region, a clear understanding of which interaction is occurring under specific circumstances remains elusive.

In the *mdm*‐mouse model, an 83‐amino acid deletion spans part of the I83 domain and the PEVK region, disrupting the I83 domain's structure and preventing the binding of the N2A region to F‐actin (Hayashi et al., 2008). This deletion causes the muscular dystrophy with myositis (*mdm*) phenotype (Figure 1) (Hackman et al., 2002), shortening the I83 domain (Garvey et al., 2002) and disrupting the p94 binding site that spans I82 and I83 (Figure 1) (Huebsch et al., 2005). While passive stretch is unaffected, the 3‐ to 4‐fold increase in force typically observed during active stretch is absent in *mdm* muscles (Powers et al., 2016), suggesting that the deleted sequence plays a critical role in titin's contribution to active force (Powers et al., 2016). Since binding sites within the N2A region often span two domains, further investigation of the I83 domain, particularly in tandem with the neighboring I82 domain, is necessary to understand how this portion of the N2A region responds to calcium and how the adjacent site impacts domain stability.

Tandem pairs of immunoglobulin domains in titin have been studied in both the A‐band and the I‐band, with varied outcomes. Jane Clarke's lab demonstrated that the presence of a neighboring domain increased stability (larger ∆G_unfolding_) and decreased the unfolding rate in tandem Ig domains (Randles et al., 2008). Another study showed that tandem pairs of Ig domains in the A‐band of titin behaved differently: one pair (A164‐A165) acted as a cooperative unit with enhanced stability, while another pair (A168‐A169) exhibited independent folding behavior (Steward et al., 2012). These differences were attributed to distinct patterns of domain/domain interface interactions (Steward et al., 2012). In contrast, tandem Ig domains in the I‐band, which fold independently, displayed significant variations in folding rates, with no observed enhancement in stability due to their neighboring domains (Scott et al., 2002). This suggests that this region behaves as the “sum of its parts” (Scott et al., 2002). Work from the Fernandez lab has shown that repeated mechanical unfolding of tandem domains can lead to misfolded structures involving the two neighboring domains (Oberhauser et al., 1999), highlighting the complexity of refolding multidomain proteins.

Independence may be advantageous in the extensible region of the I‐band, whereas cooperativity may benefit tandem domains that form binding sites within the N2A region. In the context of this manuscript, we are defining cooperativity as the degree of influence between the various structural elements in the protein. Our previous study revealed substantial differences in both the chemical stability (as measured using increasing urea as a denaturant) and folding rates for the I82 and I83 Ig domains (Kelly et al., 2020). Specifically, the I82 domain exhibited nearly twice the chemical stability of I83 (6.4 kcal/mol to 3.31 kcal/mol respectively), and its folding kinetics were an order of magnitude slower than I83 (8.1 to 0.56 s^−1^, respectively) (Kelly et al., 2020). Interestingly, the stability of the I83 domain increased at 50 μM Ca^2+^ (3.31 kcal/mol in the absence of Ca^2+^ to 4.86 kcal/mol in the presence of Ca^2+^), but similar stabilization was not observed in the I81 or I82 domains. NMR studies on the I83 domain indicated a high level of perturbation near the N‐terminal linker to I82 in the presence of calcium (Kelly, Pace, et al., 2021) and mutations in this region resulted in a loss of the calcium‐induced stabilization. This suggests that calcium might affect the domain/domain interface, potentially altering the cooperativity between these tandem domains. Even small changes in domain conformation or hinge angles could sterically alter binding sites on titin (Zacharchenko et al., 2015), which might be important given that the p94 binding site spans I82 and I83 (Hayashi et al., 2008).

In this study, we compare the ∆*G*_unfolding_ of the tandem I82‐I83 construct to an equimolar mixture of the two domains to determine whether they behave independently or as a cooperative unit. These experiments were carried out at both 50 μM Ca^2+^ and calcium‐free conditions to evaluate any calcium‐dependent changes in stability, cooperativity, or structure. We also report the NMR structure of the tandem I82‐I83 construct and show that the domain contacts along the domain interface are different in solution compared to the crystal structure of this region. We postulate that the differences in the interfaces may represent different functional states. These findings provide further insight into the role that the N2A region plays in titin function.

## 2 RESULTS

### 2.1 Cooperativity in the tandem construct is enhanced by calcium

Work from our lab has demonstrated differences in the chemical stability, folding kinetics, and calcium sensitivity of three immunoglobulin domains (I81, I82, and I83) distal to the insertion sequence in the N2A region (Kelly et al., 2020). These differences are thought to play a role in maintaining binding sites and modulating force in the N2A region. The tandem I82‐I83 domains form one of the known binding sites for the p94 protease (a muscle‐specific calpain protease) in the N2A region (Hayashi et al., 2008). This binding site is not functional in *mdm*‐mice, where an 83‐amino acid deletion disrupts the structure of the I83 domain (Huebsch et al., 2005). This disruption suggests that the proper structure of both domains is necessary for the binding site to be functional. Determining the stability of the tandem I82‐I83 Ig domain pair will provide key insights into the stability of the binding site they form. Furthermore, understanding how interdomain interactions affect the folding behavior of the tandem domains will shed light on how the p94 binding site is maintained. Lastly, exploring the impact of calcium on the stability of the tandem domains will provide important information about how the binding site might be altered in active muscle.

Previous study in our lab had shown that the I83 domain, with a stability of 3.31 kcal/mol, is less stable than most Ig domains in titin, while I82, with a stability of 6.24 kcal/mol, is more stable than I83 but less stable than many of the domains in the distal Ig region of titin (Carrion‐Vazquez et al., 1999; Garcia et al., 2009; Kelly et al., 2020). Despite the I82 domain being almost twice as stable as I83, both domains exhibited similar *m*‐values (1.2 kcal/mol for I82 and 1.0 kcal/mol for I83) in our previous studies (Kelly et al., 2020). The *m*‐value is a measure of how cooperative the unfolding process is for a protein. The previously measured values suggest that while there is a difference in the chemical stability of I82 and I83, they unfold with similar degrees of cooperativity. Interestingly, in the presence of calcium, the stability and cooperativity of the individual I83 domain increased significantly (Kelly et al., 2020). Based on our previous results, there were two possible predicted results: unfolding is cooperative and there is one transition, or the two domains unfold independently, resulting in two distinct transitions.

**FIGURE 2:**
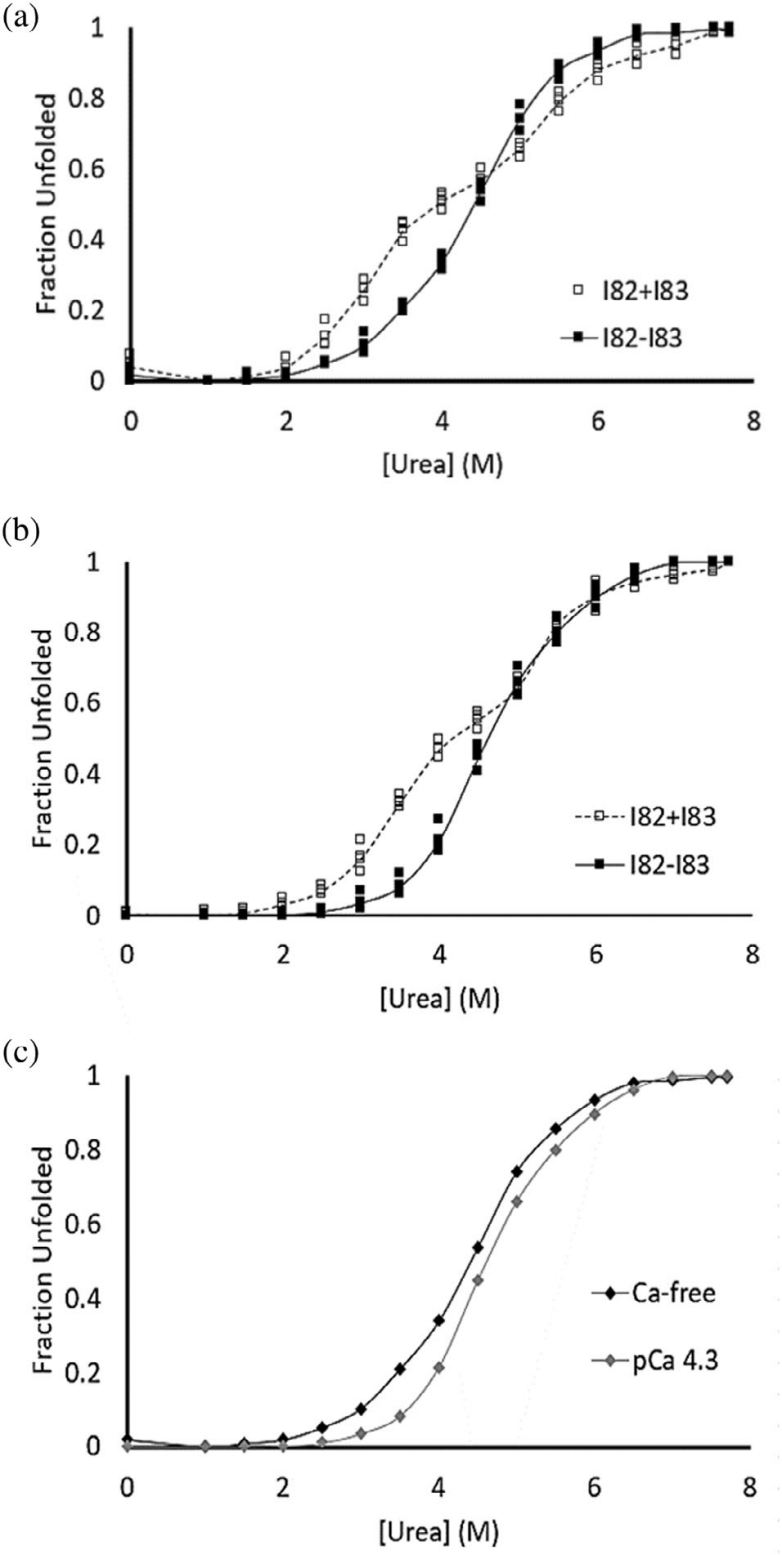
Equilibrium assays yield more cooperative unfolding for tandem I82‐I83 than for I82 + I83. Average of three trials were plotted on each graph. (a) The stability in the absence of calcium of the tandem I82‐I83 (solid black line, filled circles) yields a cooperative, two‐state unfolding event whereas the equimolar mixture of I82 + I83 (dashed line, open circles) fits a three‐state unfolding model with an intermediate at 3.5 M urea. (b) In the presence of calcium (50 μM Ca^2+^), the tandem I82‐I83 (solid black line, filled circles) unfolds in a cooperative, two‐state model and the equimolar mixture of I82 + I83 (dashed line, open circles) fits a three‐state unfolding model with an intermediate at 4.0 M urea. (c) The tandem I82‐I83 unfolds at higher concentrations of urea and demonstrates a more cooperative unfolding with a steeper slope for the transition in the presence of 50 μM Ca^2+^ (gray line) than in the absence of calcium (black line).

Chemical denaturation using urea was used to compare the equilibrium stability for an equimolar mixture of I82 and I83 (I82 + I83) with the tandem I82‐I83 domains (Figure 2 and Table 1). These studies were conducted in a buffered saline solution (20 mM HEPES, 138 mM KCl, 12 mM NaCl, pH 7.4) with varying concentrations of urea. The unfolding curve for the I82‐I83 domain in the absence of calcium was consistent with a two‐state model where the domains cooperatively unfold rather than unfold independently. If this model is correct, it would suggest that the interface between the two domains helps to stabilize the I83 domain while destabilizing the I82 domain, based on the previously reported ∆*G*_unfolding_ for the individual domains (Kelly et al., 2020).

**TABLE 1:** Equilibrium data for tandem and individual domains demonstrates cooperative.

	Ca‐free		50 μM Ca^2+^	
	∆*G* (kcal/mol)	*m*‐value (kcal/mol)	∆*G* (kcal/mol)	*m*‐value (kcal/mol)
I82	6.24 ± 0.44	1.21 ± 0.04	6.42 ± 0.14	1.25 ± 0.03
I83	3.31 ± 0.30	1.01 ± 0.08	4.86 ± 0.05	1.30 ± 0.03
${\bar{x}}_{I82,I83}$	4.78 ± 0.35	1.11 ± 0.06	5.65 ± 0.10	1.28 ± 0.03
I82 + I83	4.60 ± 0.09^a^	1.12 ± 0.03^a^	5.75 ± 0.21^a^	1.32 ± 0.04^a^
I82–I83	4.93 ± 0.14^b^	1.24 ± 0.05^b^	5.96 ± 0.27^b^	1.44 ± 0.09^b^

*Note*: Unfolding in tandem and enhanced stability at 50 μM Ca^2+^. The intermediate state in three‐state unfolding creates a barrier between the native and unfolded states, meaning that the overall reaction is not a simple thermodynamic equilibrium, but two different equilibria (Harder et al., 2004). The reported single free energy value for the overall process from I82 + I83 is a sum of two transitions and not a true Gibbs free energy value by definition because it is not describing a single transition between two states, but it allows for comparison with the I82‐I83 construct.

^a^ Free energies and m‐values for the equimolar mixture of the two domains (I82 + I83) were best fit using three‐state unfolding.

^b^ Free energies and m‐values for the tandem I82‐I83 pair were best fit with a two‐state system better than a three‐state system; thus, Δ*G*, and m‐values of I82‐I83 are assumed to be “true” values.

An alternative model that needs to be considered is that the measured shifts in the Center of Mass are the additive signal from each individual domain and there is no cooperativity between the domains. To test this, we determined the unfolding curve for an equimolar mixture of I82 and I83 (I82 + I83). Unlike the I82‐I83 construct, the I82 + I83 mixture displayed two distinct unfolding transitions. The I82 + I83 data was fit as two separate unfolding events and the plateau between the two unfolding curves was used as the upper baseline for the lower urea unfolding event and the lower baseline for the higher urea unfolding event, using the approach developed by Harder et al. (Harder et al., 2004) (Figure 2a and Table 1). The plateau between the two transitions is hypothesized to represent a state where the I83 is predominantly unfolded and the I82 domain is folded. The presence of the two distinct states from the individual domains provides support for the model of cooperative unfolding since we would expect to see the two unique transitions if there were no interaction between the two domains during unfolding.

When these stability tests were repeated at 50 μM Ca^2+^, the tandem I82‐I83 construct again demonstrated two‐state unfolding, with enhanced stability and cooperativity in the presence of calcium. In these conditions, unfolding began at higher urea concentrations, and the transition between folded and unfolded states had a steeper slope (Figure 2b,c and Table 1). The calcium‐induced stabilization of the tandem construct was expected, given the known calcium response of the individual I83 domain (Kelly et al., 2020). The tandem construct also exhibited a higher *m*‐value in the presence of calcium, indicating that calcium not only prevents unfolding but also compresses the range of denaturant needed to induce unfolding, suggesting a higher degree of cooperativity during unfolding in the presence of Ca^2+^. Therefore, calcium enhances both the stability and the cooperativity of the tandem I82‐I83 construct.

Free energies and *m*‐values for I82 + I83 and I82‐I83 were compared to those for the individual domains. The equilibrium data for both the I82 + I83 and the tandem I82‐I83 construct closely match the theoretical average of the individual I82 and I83 (${\bar{x}}_{I82,I83}$). However, the tandem pair showed increased cooperativity, as evidenced by the higher *m*‐value. Calcium is known to enhance the stability of I83 (Kelly et al., 2020), and a similar effect was observed for both I82 + I83 and I82‐I83 at 50 μM Ca^2+^.

### 2.2 Structural changes induced by calcium

Structural analyses were completed on the tandem construct in both the presence and absence of calcium, using tryptophan fluorescence to monitor local environmental changes and circular dichroism to examine changes in the overall secondary structure of the tandem construct at 50 μM Ca^2+^. Samples were incubated in 50 μM Ca^2+^ or 1 mM EDTA for 1 h at room temperature before scanning. Denaturation was initiated with 8 M urea to compare unfolded structures. A comparison of the min (folded) and max (unfolded) CoM values for each fluorescence emission spectrum was performed. Minimum CoM values were found between 0 and 1 M urea and maximum CoM values were found at 7.7 M urea. The values were normalized to the minimum and maximum for each experiment and plotted ±SD (Figure S1a). The normalized CoM for the folded construct in the presence of 50 μM calcium was 7.8 ± 0.5% lower than in the absence of calcium. A paired t‐test was performed (*N* = 4) comparing minima and maxima in each experiment, which yielded a statistically significant (*p* = 0.0024) difference between the folded samples with and without 50 μM calcium. There was no significant difference in the CoM of the unfolded construct when compared in this way. This suggests that calcium induces a shift to a more hydrophobic environment for one or more of the tryptophan residues in the structure. These results align with our recent NMR study reporting perturbations near tryptophan 42 in the I83 domain (Vivian & Callis, 2001). Circular dichroism of the native structure showed a slight increase in β‐sheet content and a decrease in molar ellipticity at 50 μM Ca^2+^ (Figure S1b), further suggesting the potential that calcium induces subtle conformational changes to the native state of the tandem I82‐I83.

### 2.3 NMR characterization & assignment

**FIGURE 3:**
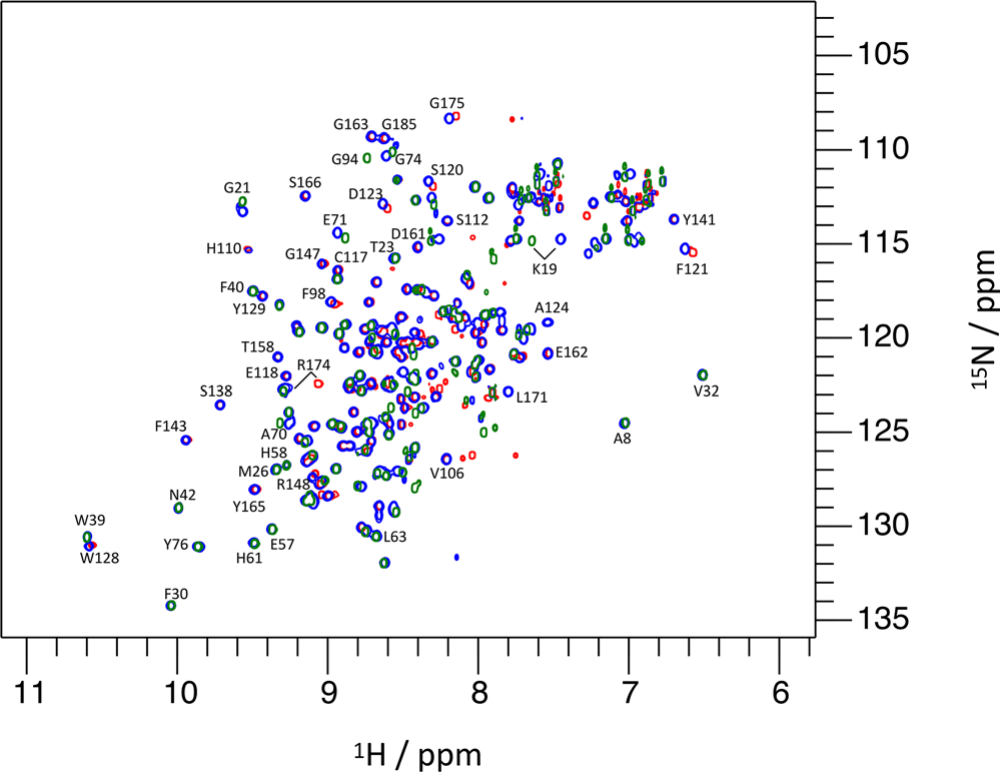
Superimposed 2D ^15^N HSQC spectra of titin domain I82 (green), I83 (red) and the tandem of I82‐I83 (blue). Selected, well resolved residues are labeled.

**FIGURE 4:**
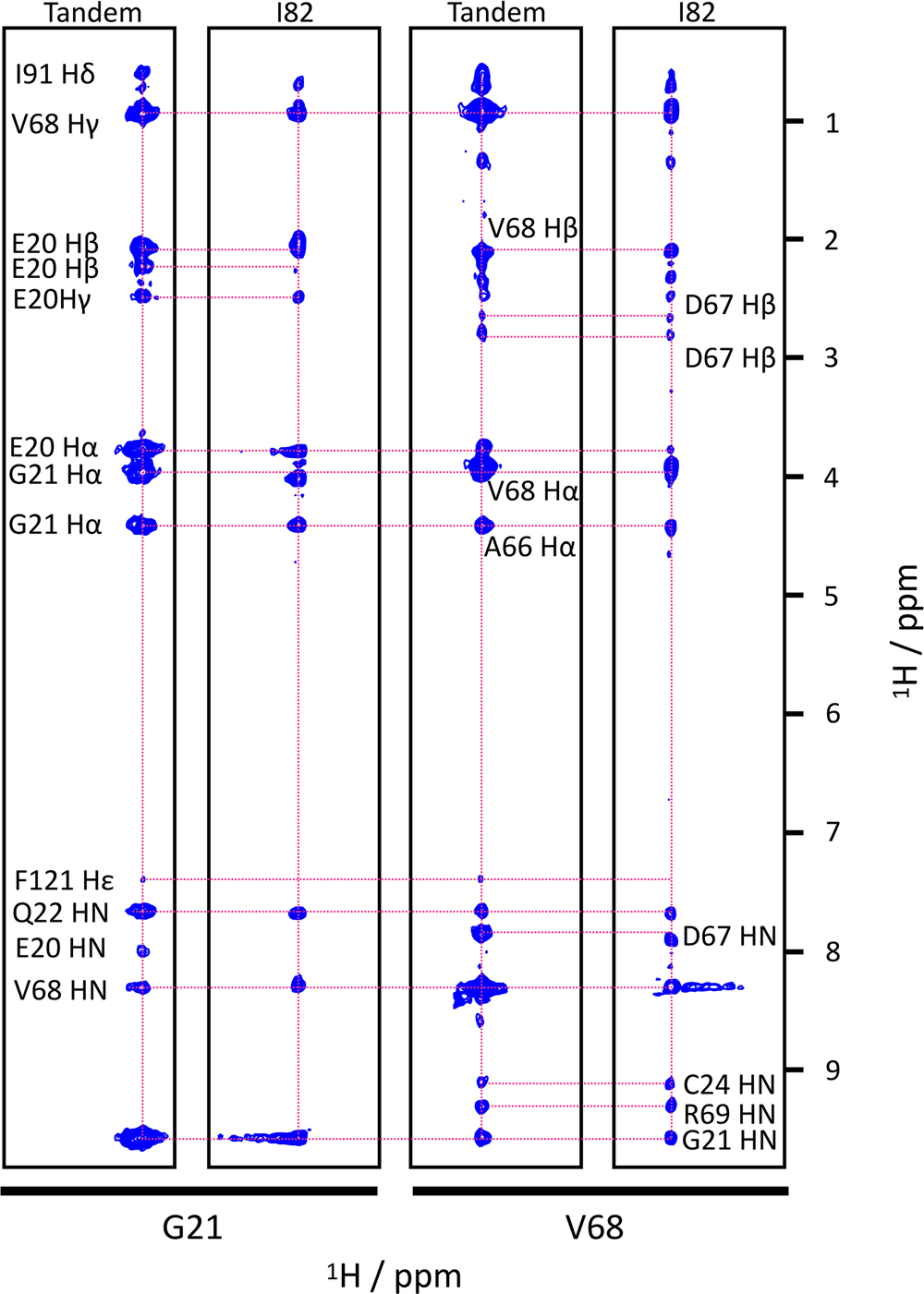
Strips of 3D NOESY‐HSQC spectra of the I82‐I83 tandem and I82 alone for residues G21 and V68 to indicate the changes in chemical shifts in the domain–domain interface and the additional NOE (to F121 He) that connects the two domains in the interface. Selected cross peaks from nearby resonances are indicated.

**FIGURE 5:**
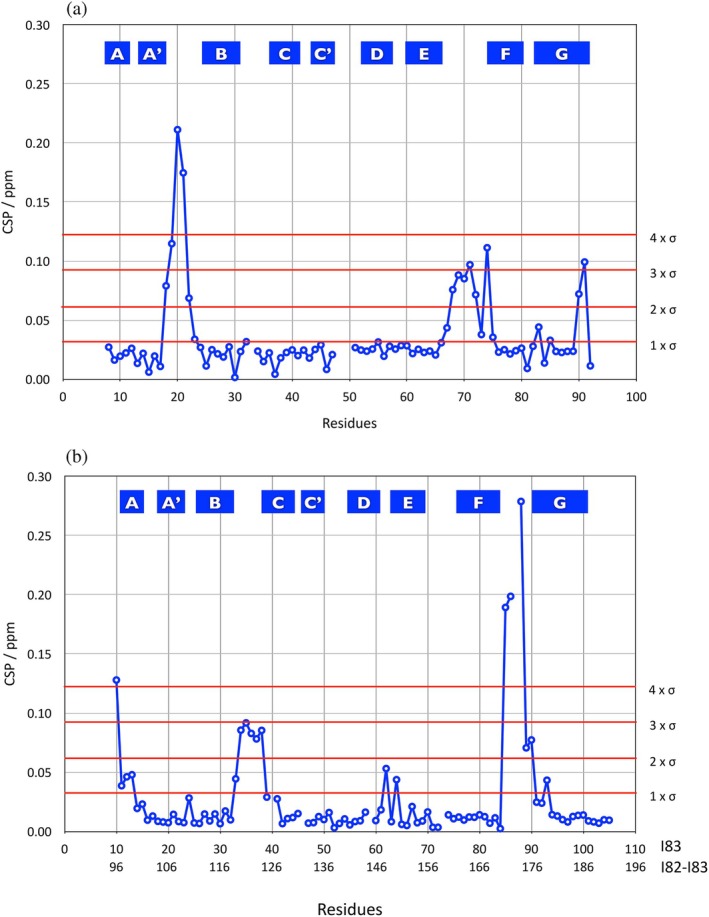
Chemical differences of equivalent residues in the individual domains I82/I83 compared to the tandem. (a) chemical shift differences for I82. (b) chemical shift differences for I83. The standard deviation of the whole set of differences is shown in red lines as integer multiples.

Backbone assignments for the I82‐I83 tandem were obtained initially from the assignments of the individual domains in the BMRB (I82: 34980/34981 and I83: 50015). Spectra of the individual domains were globally very similar to the spectrum of the tandem (Figure 3). The largest changes were observed for the N‐terminus of I83 and the C‐terminus of I82. More modest chemical shift perturbations were observed in the contact regions of the two domains. To resolve ambiguities in the assignment a 3D ^15^N NOESY experiment was recorded (Figure 4). As a result, a complete backbone and partial side chain proton assignment could be obtained for all residues in the I82‐I83 tandem. The chemical shift perturbations (CSP) showing differences between the individual IgI domains and the tandem are shown in Figure 5 identifying clearly regions of contact between the two domains: most involved (CSP >0.1) are residues in the A'B and the EF loop of I82 and the BC and the FG‐loop of I83.

### 2.4 Tandem structure

**FIGURE 6:**
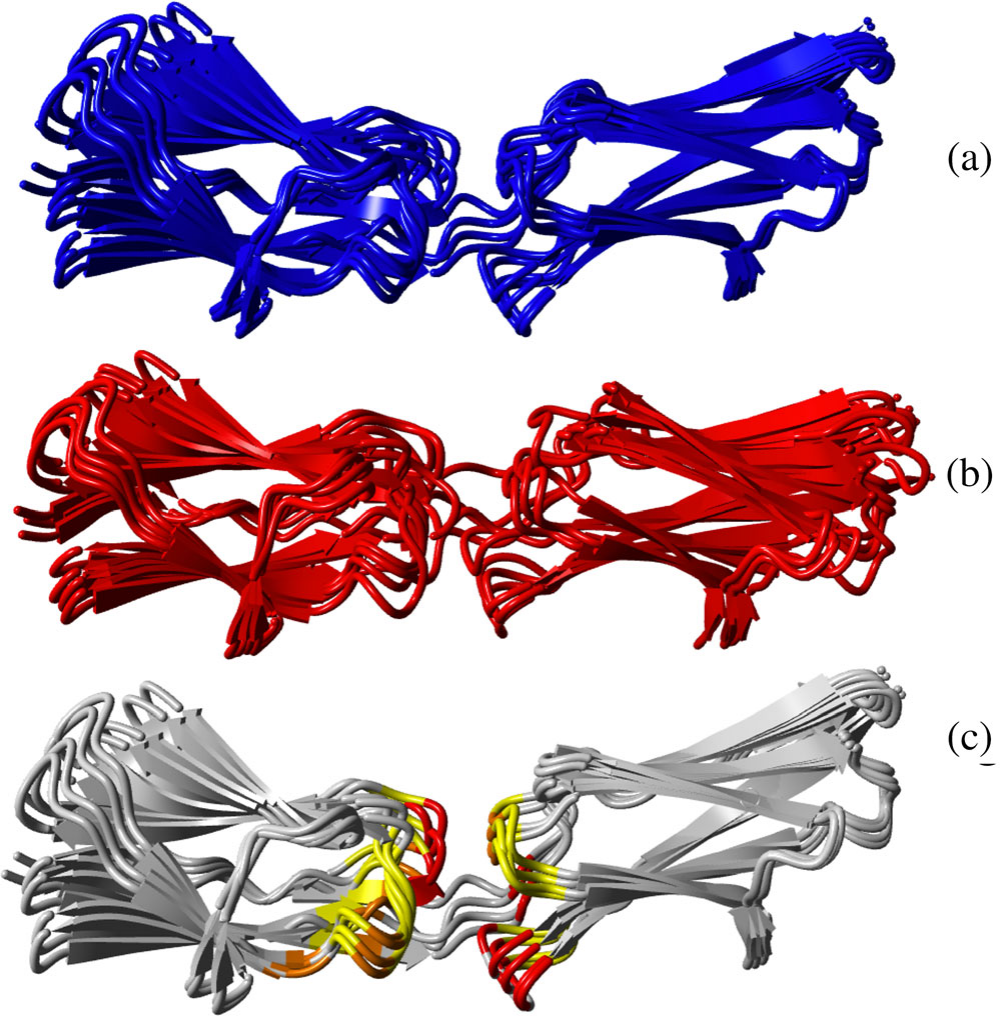
Solution structure family of the I82‐I83 tandem. Domain I82 is left, domain I83 is right. Separately superimposed are the top four structures from the best clusters for (a) I82OUT and (b) I82IN. (c) Structure family for I82OUT with CSP values from Figure 7 shown in color (2 * σ = yellow; 3 * σ = orange; 4 * σ = red).

A model for the solution structure of the I82‐I83 tandem was calculated using the following experimental NMR data: chemical shift perturbations (CSP), inter‐domain NOE distance constraints, and residual dipolar couplings (RDC) and the experimental structures of the independent domains. Both conformers of I82 (PDB ID: 9IBI and 9IBK) were used as starting models in separate calculations. Structure calculations using the different conformers of I82 resulted in different numbers of HADDOCK clusters; I82IN led to 6, I82OUT to 3 (Figures 6, S4 and Tables 2 and 3). Closer inspection shows that the best clusters of each calculation (I82IN cluster 2: heavy atom RMSD = 1.61 Å; I82OUT cluster 1: heavy atom RMSD = 1.24 Å) are very similar (Overall heavy atom RMSD amongst all eight structures in the two clusters combined = 1.84 Å; heavy atom RMSD between the two closest structures of each cluster = 0.81 Å) so that all subsequent analysis was carried out with a representative of I82OUT cluster 1 (see Figure 6 and S4) as this had the best overall restraint energy, best RMSD/FCC parameters as well as the vast majority of the total calculated structures (476 out of 500, see Table 3). As such, it best represents the experimental constraints used in the modeling.

**TABLE 2:** Structure quality statistics for the HADDOCK structure calculation using the I82IN conformer.

Cluster	1	2	3	4	5	6
Score	44.7	45.4	78.2	71.7	49.2	70.2
Number	396	45	24	9	9	4
RMSD	11.1	2.3	11.5	2.5	1.6	2.5
Restraint	268.8	161.0	344.7	297.2	126.0	212.8
Solvation	6.8	8.2	0.8	5.4	11.5	6.8
vdW	−15.0	−10.5	−14.1	−8.0	−21.8	−8.6
Z‐score	−1.1	−1.1	1.3	0.9	0.7	0.7

*Note*: Cluster: Number of the cluster. Score: Haddock pseudo energy score summarizing the geometrical quality of the structure with violation of experimental constraints. Number: Number of structures in each cluster (out of a total of 500). RMSD: RMSD value of cluster average to the lowest score structure. Restraint: Violation of experimental restraints. Solvation: Solvation energy. vdW: vdW energy (essentially clash score, the lower the better). Z‐score: How many standard deviations from the average this cluster is located in terms of score (the more negative the better).

**TABLE 3:** Structure quality statistics for the HADDOCK structure calculation using the I82OUT conformer.

Cluster	1	2	3
Score	29.6	44.4	71.1
Number	476	11	10
RMSD	1.5	1.6	3.7
Restraint	163.8	212.2	205.8
Solvation	6.5	7.1	3.3
vdW	−19.1	−19.9	−6.0
Z‐score	−1.1	−0.2	1.3

*Note*: For details see Table 2.

**FIGURE 7:**
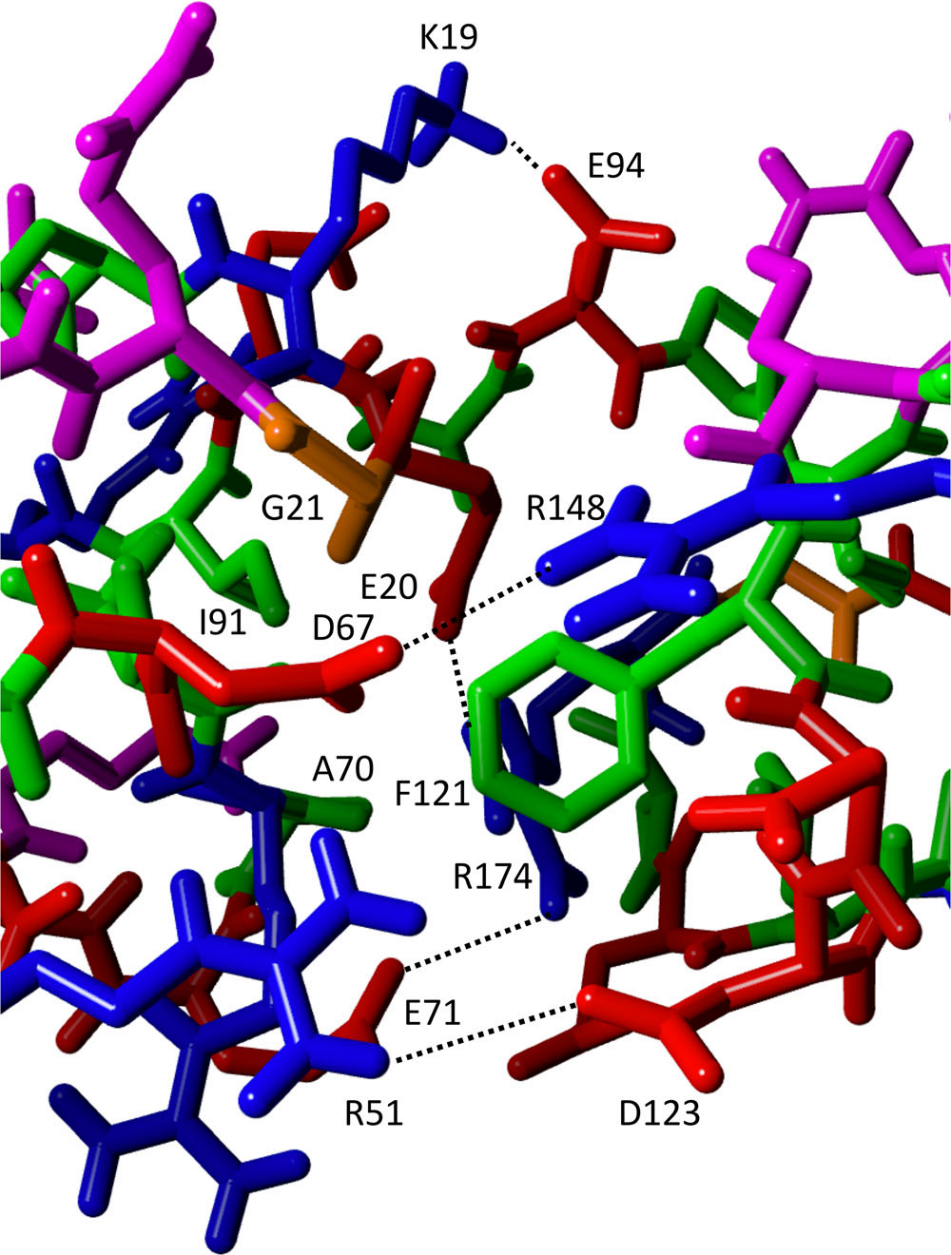
The interface of domain I82 (left) and I83 (right) in the solution structure of the tandem. Heavy atoms and polar hydrogens are shown. Amino acid types are indicated in color: Green: Hydrophobic; Magenta: Hydrophilic; Red: Negatively charged; Blue: Positively charged; Orange: Glycine. Interdomain hydrogen bonds/salt bridges are indicated by lines. Selected residues are labeled.

Overall, the solution structure of the I82‐I83 tandem is quite extended with only the C‐terminal loops of I82 and the N‐terminal loops of I83 making contacts with the partner domain. The angle between the principal axes of both domains is ~23° and the twist around the common principal axis (i.e., the angle between the β‐sheets) is ~43°. The main contacts between the domains are between the A'B‐loop of I82 and the BC‐loop of I83 as well as the EF‐loop of I82 and the FG‐loop of I83 in addition to contacts of both loops with linking residues (A93‐I96) between the domains (see Figure 6). The solvent‐exposed surface buried by the tandem interaction is 322 Å^2^. The main stabilizing contacts are salt bridges between D67‐R148, R51‐D123, E20 and E71 with R174 and K19 with E94 (Figure 7). Some hydrophobic clusters are seen as well, such as A70, I91, A93 packing on the sidechain of R174. Likewise, F121 makes contacts with the backbone of G21 and the aliphatic side chains of L171, I96 and the aliphatic side chain portion of E20. The close contact of the aromatic ring with E20 is confirmed experimentally by the changes of the chemical shift of the side chain Hβ or Hγ resonances of E10 (Figure 4) which are shifted by ~0.1–0.2 ppm in good agreement with being close to an aromatic ring. In contrast, the ring protons of F121 do not really show any change because being near aliphatic protons normally does not affect aromatic protons. Interestingly, any other residue in place of G21 would possibly cause a clash with F121 so that a glycine in this key position might be important to allow the compact interface observed in solution. Coincidentally, this residue is highly conserved and is part of the residues that define the intermediate set of Ig domains (Harpaz & Chothia, 1994). While there are only a few distance constraints in the interface it is still well defined by the high number of RDCs which serve to position the two domains. As the relative position of the two domains can be defined by three angles and one distance even some noise in individual RDC values is well canceled out by the sheer number of them (a total of 172).

### 2.5 Comparison to X‐ray structure

**FIGURE 8:**
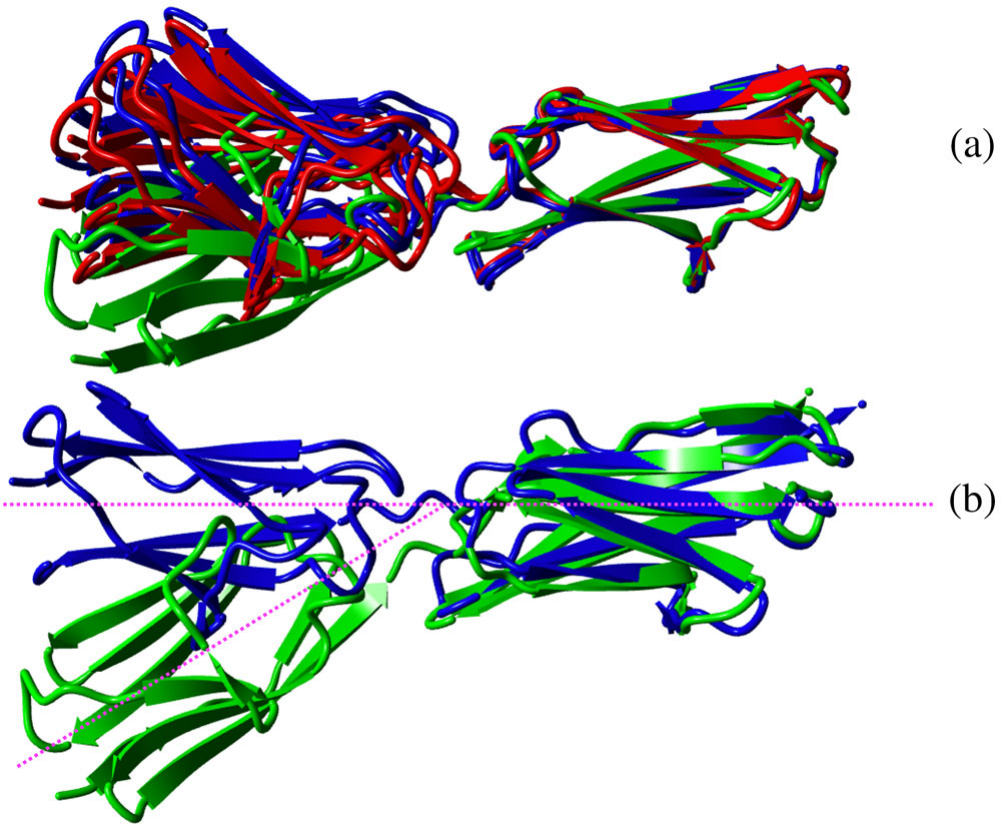
(a) Superposition of the NMR structure of the I82‐I83 tandem onto that determined by X‐ray crystallography (both clusters of the NMR structure shown as in Figure 6, that is, I82IN in red, I82OUT in blue). I82 is on the left, I83 on the right. (b) Superposition of the crystal structure (green) on the representative NMR structure (in blue, slightly rotated compared to A to make the difference in domain orientation more evident). The principal axis for the solution structure is indicated by a magenta line to illustrate the difference in domain position between the solution and the NMR structure.

The I82‐I83 interface in the crystal (PDB ID: 7AHS) (Stronczek et al., 2021) buries only 122 Å^2^, just about 1/3 of that observed in solution. The main cause of this difference is a tilt of the principal axes of the two Ig domains relative to each other by ~40° while the orientation of the planes formed by the β‐sheets in each domain is virtually identical with a rotation of only about 25° between the structures (Figure 8). Therefore, the interfaces are similar with the main difference being a greater proximity of the A'B‐loop of I82 to the BC‐loop in I83. Consequently, interactions of F121 with G21 seen in the solution structure are not present in the crystal structure. Essentially, the X‐ray structure resembles a somewhat opened version of the solution structure. It could be that the solution structure represents the tandem interface as it exists in the sarcomere in the absence of force. A small amount of force could open the interface to lead to an overall more extended structure as represented in the X‐ray structure.

It is also interesting to consider the very small, buried solvent accessible surface of the tandem interface in the crystal structure compared to the solution structure. A very small interface area combined with only a few contacts is not expected to be highly stable. In contrast, there are extensive contacts in the crystal that far outweigh the sequential interdomain interface between I82–I83. The tandem forms antiparallel dimers with an offset of one domain in the crystal structure. This allows for the creation of a ‘sticky’ end so that long ribbons are formed. Individual ribbons assemble by packing both in parallel as well as roughly orthogonally (Figure S5a). As a result, there are multiple, sizeable interfaces between neighboring molecules. On average, the antiparallel dimers bury about 1100 Å^2^ solvent accessible surface between them while the two different contacts in the overlap regions cover about 120 and close to 400 Å^2^ (see Figure S5b). In addition, there are further contacts between orthogonal ribbons so that one can conclude that the intermolecular contacts far outweigh the contacts in the interdomain interface. As a consequence, the tandem interface in the crystal could be forced to assume a more extended conformation to better fit into the ribbon arrangement in the crystal.

### 2.6 NMR relaxation studies

**FIGURE 9:**
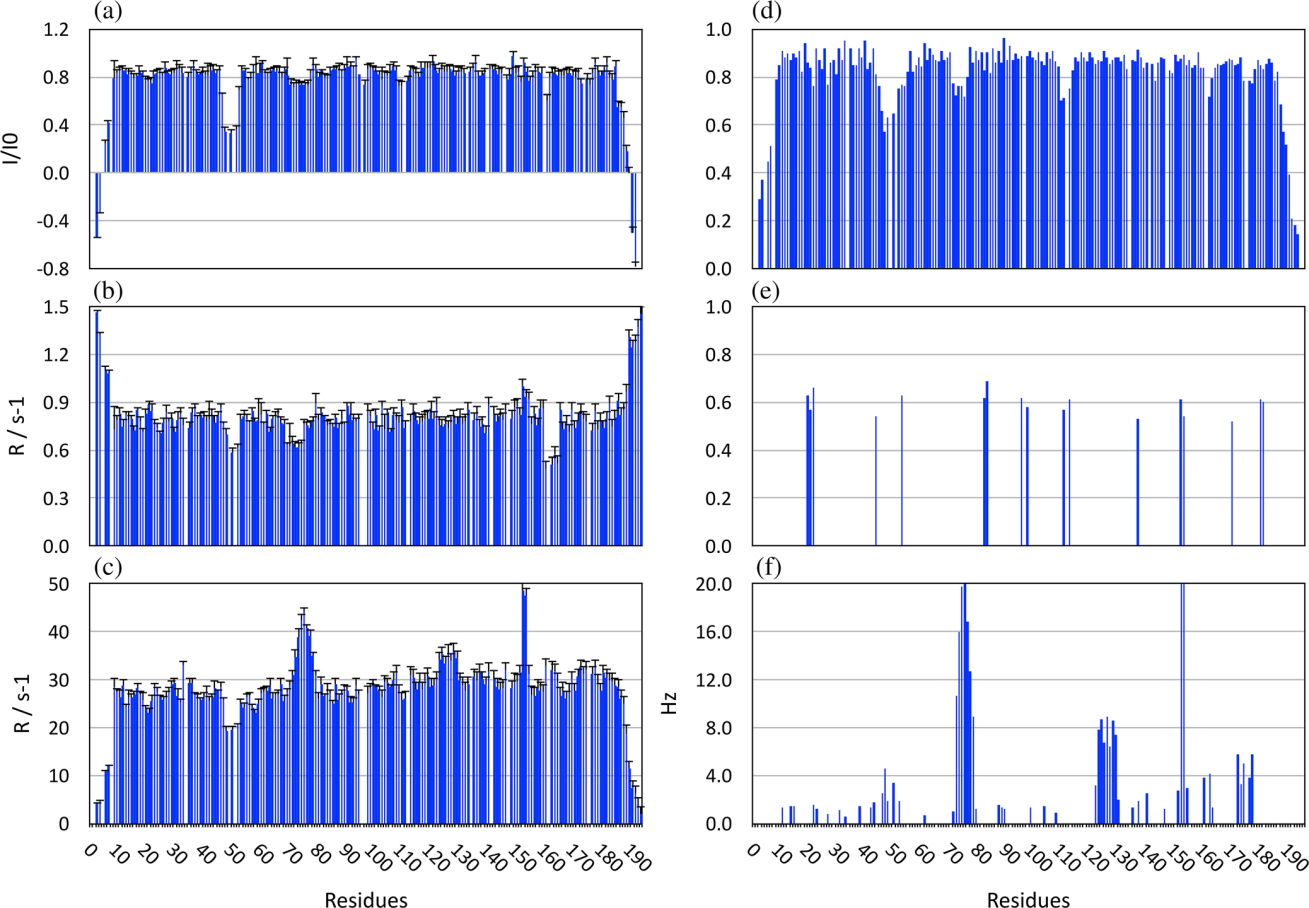
Relaxation and dynamics analysis. (a) ^1^H‐^15^N heteronuclear NOE. (b) ^15^N R1. (c) ^15^N R2. (d) Order parameter S^2^ for ps timescale mobility. (e) Order parameter S^2^_i_ for ns time scale mobility. (f) Exchange broadening contribution R_ex_ to R_2_.

Relaxation data (^15^N R_1_, ^15^N R_2_ and ^1^H‐^15^N heteronuclear NOE) were collected for the tandem protein (Figure 9a–c). Analysis of the structure of the tandem (Figure 6) using PDBinertia (Iii et al., 1991; Mandel et al., 1995) suggested a rather high level of anisotropy (relative moments along the X, Y and Z axes: 1.0:0.97:0.15) so that the usual approach for the determination of the rotational correlation (isotropic rotational diffusion) could not be used. Instead, the ^15^N R_2_/R_1_ values were fitted using R_2_R_1_diffusion (Iii et al., 1991; Mandel et al., 1995) with a model of axially symmetric rotational diffusion. The rotational correlation time was tauc = 16.2 ± 0.3 ns with a ratio of the parallel to the perpendicular rotational diffusion coefficient (D||/D⊥) of 2.69 ± 0.13. The axially symmetric diffusion tensor is in excellent agreement with the predicted inertia moments calculated from the structure with only very small rotations of θ = 0.6° and φ = 4.8°.

The individual relaxation values are fairly uniform along the whole protein with some local exceptions. Negative NOE values, high R_1_ and low R_2_ values suggested high mobility on the ps time scale at the termini while more modest fast mobility is seen for the CD loop of I82 (Q49‐V52), the EF‐loop of I82 (D72‐C78) and around H110 in the AB‐loop of I83. The Lipari‐Szabo Analysis (LSA) confirms fast local mobility in all these regions with significantly (termini) or modestly (loops) lower order parameters (Figure 9d). Significant increases in R_2_ are seen in the EF‐loop of I82 (residues A70‐Q75), the BC‐loop of I83 (S120‐A124) and residues H150 and Y151. LSA analysis shows that these are caused by slow chemical exchange with substantial line broadening of ~20 Hz in the EF‐loop of I82 and H150/Y151 in I83 and slightly lower values for the I83 BC‐loop (~8 Hz).

It is interesting to note that all regions of variability in the relaxation parameters observed for the I82‐I83 tandem are virtually identical to those observed in the individual domains (Kelly, Pace, et al., 2021). However, some differences were noted that are of some significance. The zone featuring chemical exchange most prominently in the EF‐loop of I82 is more extended and reaches into the preceding 3_10_ helix in the isolated domain while it starts a bit later in the tandem structure (D72 in the tandem vs. A70 in I82 alone). We see something similar in the BC‐loop of I83 (residues 123–128) where significant line broadening is still seen but at a much‐reduced level (~8 Hz in the tandem compared to >15 Hz in the individual domain).

The differences in exchange line broadening observed both in the EF and BC‐loop between the individual domains and the tandem can be explained by the involvement of each loop in the tandem interface. The complete BC‐loop of I83 is making extensive contacts with I82 as evidenced by the CSP values (Figures 5b and 6c). These close contacts can explain the reduction of local mobility on the μs–ms time scale. By contrast, the line broadening in the EF‐loop results from the conformational interchange between the “IN” and “OUT” conformers around residue G75 in I82. However, it appears that these are somewhat dampened in the preceding 3_10_ helix possibly because these residues form part of the tandem interface. In contrast, conformational exchange of the IN/OUT conformers in I82 as well as the dynamics around H150/Y151 in I83 continue unaffected in the tandem.

### 2.7 Calcium binding

**FIGURE 10:**
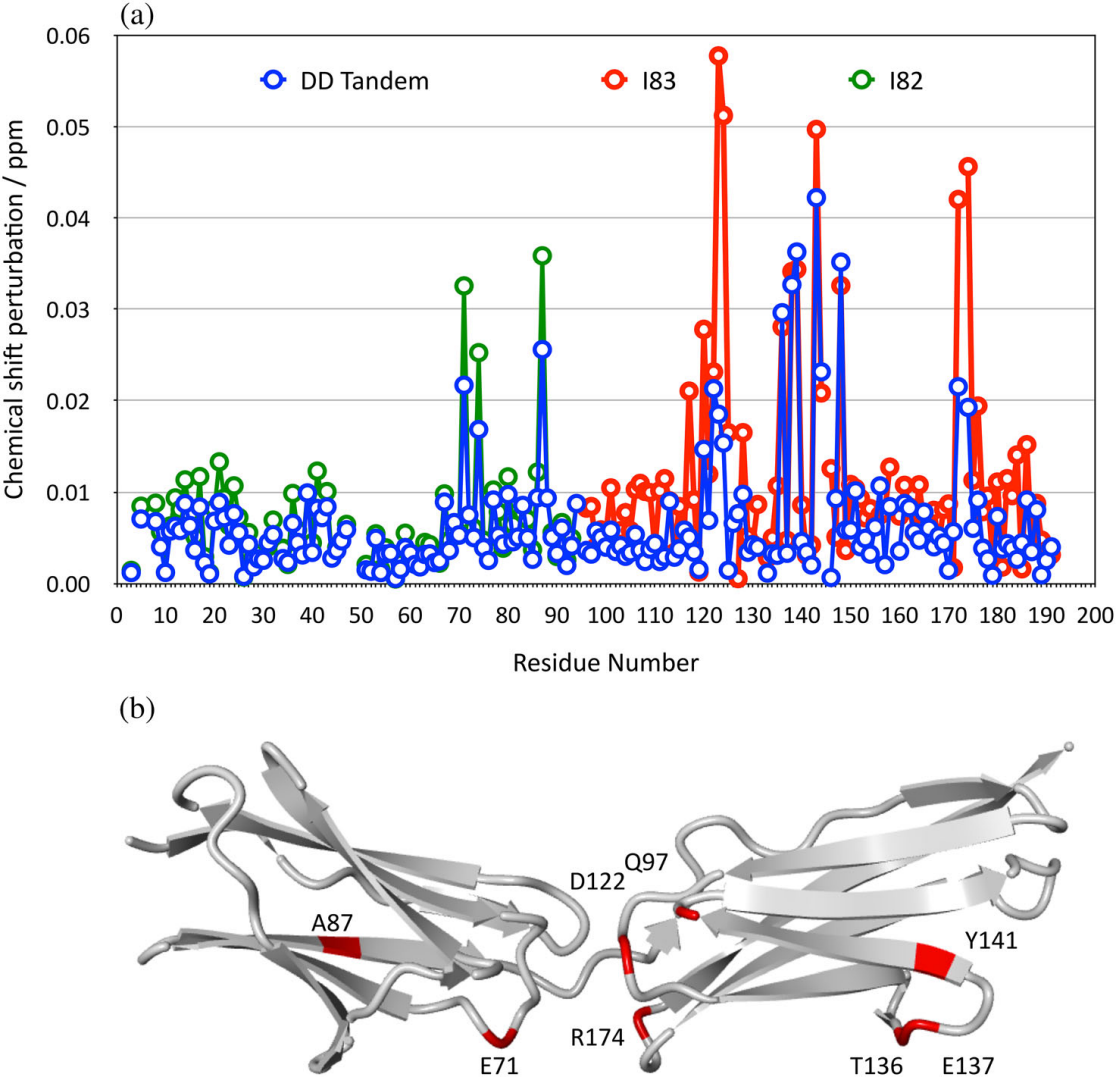
Results of NMR calcium binding experiments. (a) Chemical shift perturbations (CSP) for the addition of calcium to the I82‐I83 tandem (blue), I82 on its own (green) and I83 on its own (red) plotted against the sequence. (b) CSP values >0.02 for the tandem mapped on the structure of the tandem (red) with labels for the corresponding amino acids.

The results of the calcium binding experiment are shown in Figure 10 for the I82‐I83 tandem, I82 and I83 on their own. As for the individual domains there is virtually no effect on the line shapes of the peaks. No titration was performed for the tandem but the similarities with the CSPs seen in I83 suggest that also here the calcium binding is in fast exchange. As we only compared two spectra with and without saturating concentrations of calcium, we cannot measure affinity or stoichiometry. The changes in the CSPs of residues in the domain interface, however, suggest that the affinity in this binding site has been reduced substantially while it remains more or less the same in the 2nd binding site. For I82 we see mainly noise with modestly increased CSP values for E71, G74 and A87 both for the domain on its own as well as part of the tandem. I83 on its own shows much higher CSP values for the BC, CD and FG‐loops as reported previously (Kelly, Pace, et al., 2021). As part of the tandem I83 still has virtually identical CSP values in the CD‐loop while they are much reduced in the BC and FG‐loops.

Residue A87 in I82 is spatially close to the negatively charged residues D15 and E88, which could make weak transient interactions with calcium without the need for high‐affinity binding. A similar situation exists for D71 and G74 which are close to a group of charged residues forming salt bridges nearby. These interactions are very weak as judged by the CSPs and so are unlikely to make a noticeable contribution to the stability of I82. High concentrations of calcium could disturb these interactions, leading to CSPs nearby. The BC and FG loops of I83 are part of the tandem interface with a number of the charged amino acids involved in calcium binding in the isolated I83 making contacts with I82 involving salt bridges and hydrogen bonds, for example, D123 making a salt bridge to R51 (see Figure 7). The ability of such residues in the I82–I83 interface to contribute to calcium binding is thus much reduced. In contrast, calcium binding in the CD‐loop is fully retained in the tandem. Interestingly, all the residues implicated in calcium binding in I83 are fully accessible in the crystal structure and not involved in the tandem interface. It should be pointed out that the reduction in calcium binding at the N‐terminus of I83 is a good confirmation of the burial of important calcium binding residues in the interface as shown in the experimental structure.

## 3 DISCUSSION

The N2A region of titin is an important signaling hub that resides in the middle of the I‐band region of the sarcomere. A variety of different proteins have been shown to interact with this region but the most relevant interaction for this study is with the calpain‐3/p94 protease, whose primary binding site is the I82‐I83 domains of titin's N2A region (Hayashi et al., 2008). A deletion of 83 amino acids spanning the C‐terminus of the I83 domain and the N‐terminus of the PEVK region causes muscular dystrophy with myositis in a mouse model (Garvey et al., 2002). This *mdm*‐deletion disrupts the structure of the I83 domain and prevents normal binding of p94 in the N2A region (Huebsch et al., 2005). Although passive stretch is not affected by this deletion, the 3‐ to 4‐fold increase in force that is typically produced during active stretch is absent in *mdm*‐mice (Powers et al., 2016). Thus, maintaining this binding site for p94 is crucial for normal muscle function and understanding the molecular nature of this region can provide important insights into the nature of this interaction.

In this study, the I82‐I83 domains were examined as a tandem pair and were found to unfold cooperatively, unlike an equimolar mixture of the two domains. Both the stability and cooperativity of the tandem domain construct were enhanced at 50 μM Ca^2+^, a calcium concentration similar to that found in the active sarcoplasm (Bootman, 2012). Circular dichroism (CD) analysis also revealed a conformational change in the tandem construct in the presence of Ca^2+^. We had previously demonstrated the presence of a calcium binding site on the N‐terminal end of I83, near the I82‐I83 interface but this site is occupied by the interface of I82 with I83 in the NMR structure and shows reduced chemical shift perturbations compared to the free I83 (Fig. 10A). These results seem to be contradictory until the I82‐I83 conformation observed in the crystal structure, which is more extended than observed by NMR, is considered.

The NMR structure of the I82‐I83 tandem shows a substantially tighter interface than the crystal structure with a buried solvent accessible surface of more than 300 Å^2^ compared to just over 100 Å^2^ in the crystal structure. This difference could be due to packing forces within the crystal forcing the I82‐I83 domains into a more open conformation to fit into the 3D lattice of the crystal. This difference in domain orientation might not be significant for many multidomain proteins that don't experience mechanical force. However, these domains are within the I‐band region of titin, which is extended when a muscle is elongated and therefore both domain orientations might be significant. We propose that the domain structure observed by NMR represents the orientation of I82‐I83 when a muscle is in a relaxed state or when the muscle is undergoing a concentric (shortening) contraction. In contrast, the more extended state observed in the crystal structure represents the state when a muscle undergoes an eccentric (lengthening) contraction.

When the muscle is in a relaxed state or is shortening, titin is in a more compressed state, and the Ig domains are loosely associated together. Under these conditions, there is no force being applied to the system to promote titin forming a more extended state, so calcium‐dependent stabilization of the I83 domain is not critical for maintaining the binding site for both calpain‐3 and F‐actin. In contrast, when a muscle undergoes eccentric contraction, both the I82 and I83 domains will experience force that is applied parallel to the long axis of the Ig domain. This will force the two domains into a more extended conformation (e.g., the configuration observed in the crystal structure), exposing the calcium binding site that has been identified in the I83 domain. This would facilitate the Ca^2+^‐stabilization of the I83 domain that has previously been observed (Kelly et al., 2020; Kelly, Pace, et al., 2021).

This stabilization would be relevant when the muscle is under load since the domains would be more likely to unfold due to the applied force. When the domains are in a more extended conformation, the binding site would be accessible, allowing Ca^2+^ to bind and stabilize the I83 domain. Experiments with the *mdm* mouse model have shown that the 89 amino acid deletion results in loss of the calpain‐3/p94 binding site, presumably due to the loss of a folded I83 domain (Hayashi et al., 2008). Force spectroscopy experiments on I83 confirmed that this domain is relatively unstable relative to the other domains in N2A (Kelly & Gage, 2021), suggesting that it would unfold and mimic the *mdm* mouse under relatively low forces, resulting in the release and activation of calpain‐3 (Hayashi et al., 2008). Maintaining domain stability as I82‐I83 becomes extended would preserve the calpain‐3 binding site until higher, potentially damaging forces, are applied and calpain‐3 would be activated.

Work from our labs has shown that I82 is critical for the binding of N2A to F‐actin but the calcium dependence associated with N2A binding to F‐actin is derived from the I83 domain (Tsiros et al., 2022). Our working model is that the I80–I82 region of N2A is the minimal unit for the binding of N2A to F‐actin and that I83 can enhance this binding when it is in a folded state. The equilibrium binding constants for the various constructs have not been determined yet but the relative affinity of the I80–I82 construct is similar to the full N2A domain in the absence of calcium, consistent with this model (Tsiros et al., 2022). Similar to the interaction between calpain‐3 and I82‐I83, stabilization of the interaction between F‐actin and N2A would be more relevant during eccentric contraction than during concentric contractions.

These proposed models fit existing data but raise several important questions that remain to be answered. First, what is the domain orientation of I82‐I83 when bound to either calpain‐3 or F‐actin? This is important to determine when the calcium binding site on I83 is accessible. Second, is I82‐I83 capable of binding calpain‐3 and F‐actin simultaneously or are they mutually exclusive? If they are mutually exclusive, what are the conditions that promote the binding of each domain? If, however, I82‐I83 can bind both proteins, how does that influence the binding affinity? Answering this second set of questions will help provide important insights into titin's role in active muscle.

## 4 CONCLUSION

The results of this study demonstrate that the interaction between the I82 and I83 may play a significant role in N2A's interactions with both calpain and F‐actin. There are differences in the interfaces between the crystal structure and the solution structure, which hint at the possibility of two states: a collapsed state and an elongated state. The collapsed state, observed in the NMR structure could represent the interface when the titin is not being stretched while the state observed in the crystal structure would represent when the titin is being stretched. We proposed that in the collapsed state, the I82 domain stabilizes the I83 domain through domain–domain interfaces. However, when the titin is stretched, this interface is disrupted and calcium can bind to the I83 domain, stabilizing it. Further work is necessary to validate this model, but it is consistent with current data.

## 5 METHODS

### 5.1 Reagents

Chemicals were obtained through standard chemical suppliers such as Fisher Scientific. The plasmids were generated using an N2A‐PEVK titin template with the Champion pET151 Direction TOPO Expression Kit (Invitrogen) and the Phusion Site‐Directed Mutagenesis Kit (ThermoScientific). Sequences were verified for all constructs using Sanger Sequencing. The NCBI sequence for the N2A isoform of titin (NP_035782) was used as the reference individual and tandem domain sequences:

I82‐I83: IEPA**W**ERHLQDVTLKEGQTCTMTCQFSVPNVKSE**W**FRNGRVL

KPQGRVKTEVEHKVHKLTIADVRAEDQGQYTCKHEDLETSAELRIEAEPIQFTKRIQNIVVSEHQSATFECEVSFDDAIVT**W**YKGPTELTESQKYNFRNDGRCHYMTIHNVTPDDEGVYSVIARLEPRGEARSTAEL

*Tryptophan residues are indicated in bold*.

### 5.2 Expression and purification of Ig domains

Both the two individual domains (I82 and I83) were expressed and purified using the following protocol: Expression plasmids were transformed into chemically competent *Escherichia coli* BL21 (DE3) cells and grown overnight at 30°C. Autoinduction media cultures were inoculated with 0.5% (v/v) of the overnight cultures and grown at 30°C for 16–18 h while being shaken at 230 rpm. I83 mutant constructs were produced using site‐directed mutagenesis (ThermoFisher) and designed clones, which were expressed in the same manner as the individual I82 and I83 domains. The tandem I82‐I83 construct followed a similar protocol but was transformed into Escherichia coli BL21 (pLysS) cells. Autoinduction media cultures for I82‐I83 were grown at 20°C for up to 24 h. All bacterial cultures were harvested by centrifugation and resuspended in lysing buffer containing IMAC wash buffer (25 mM imidazole, 250 mM NaCl, 50 mM K_2_HPO_4_), with lysing reagents: 40 μg/mL DNase, 10 mM MgCl_2_, 200 μg/mL lysozyme, 1% Triton X‐100. The cells were lysed using a combination of chemical lysing, freeze–thaw, and French press. The suspension extracted from the French press was spun at 10,000 rpm for 20 min at 4°C. The resulting supernatant was passed over a GE HisTrap HP column (GE Healthcare, immobilized metal affinity column (IMAC)) using an imidazole gradient from 50 to 200 mM imidazole to purify the expressed Ig domain. Fractions containing the protein of interest were exchanged into IMAC wash buffer (25 mM imidazole, 250 mM NaCl, 50 mM K_2_HPO_4_) and incubated with 1% TEV protease at 4°C for 48 h to cleave off the HIS‐tag. The IMAC purification was repeated following TEV cleavage with the protein without the HIS‐tag eluting in the wash. The Ig∆HIS protein was further purified by size exclusion chromatography (SEC) on a Superdex‐200 analytical column using Ig storage buffer (20 mM HEPES, 138 mM KCl, 12 mM NaCl, pH 7.4) as the mobile phase. Purity was assessed by SDS‐PAGE and SEC. Fractions were pooled and flash‐frozen in 20% glycerol to prevent aggregation during the freezing process and stored at −80°C. Protein quantitation was measured by Bradford assay.

### 5.3 Chemical stability

Chemical stability was measured using a similar approach as was used previously for the individual Ig domains (Kelly et al., 2020). Purified I82‐I83 in tandem was diluted to a final concentration of 100 μg/mL in storage buffer (±50 μM Ca^2+^) with varying urea concentrations (0–7.8 M urea). Equimolar samples of the individual domains (~50 μg/mL each) were pooled to test the stability of the mixture I82 + I83. All samples were incubated for 1 h at room temperature (~22°C) prior to data collection. Samples were analyzed in a quartz 96‐well plate. Samples were excited at 280 nm and the emission spectra were collected in 1 nm steps from 300 to 450 nm at 20°C using a SpectraMax M3 plate reader (representative raw data shown in Figure S2). The Center of Mass (CoM) of each spectrum was calculated using Equation (1), where *I* is intensity and $\bar{v}$ is the wavenumber:

(1) $$\mathrm{CoM}=\frac{\Sigma \left(\bar{v}\cdot I\right)}{\Sigma I}$$

CoM was plotted as a function of urea concentration and fit using linear extrapolation to determine the ∆*G*_unfolding_ using Equation (2):

(2) Keq=e−∆G+mxRT

where *x* is the concentration of urea. Upper and lower baselines were established for the fit by plotting the Center of Mass vs. urea data and then choosing 3–4 points before or end of the transition and fitting a line to those points (Figure S3). Equation (2) was used for the two‐state fit to calculate the fraction unfolded across all urea concentrations using Equation (3).

(3) fu=KeqKeq+1

The free energy and *m*‐value is optimized by minimizing the error between the actual Center of Mass and the calculated Center of Mass, which is determined using Equation (4), where LI = lower baseline intercept, LS = lower baseline slope, US = upper baseline slope and UI = upper baseline intercept.

(4) $${\mathrm{CoM}}_{\text{calc}}=\begin{array}{l} \mathrm{LI}+\mathrm{LS}*\text{[urea]}+f_{u}*(\mathrm{UI}+\mathrm{US}*\text{[urea]}-\mathrm{LI}\\ +\;\mathrm{LS}*\text{[urea]}) \end{array}$$

For the three‐state fit, the intermediate plateau region was estimated and used as the upper limit of the first sigmoidal curve and the lower limit of the second sigmoidal curve, and the two were added together. A comparison of a two‐state and a three‐state unfolding model was applied to stability data and the folding model of best fit was applied to the system (Harder et al., 2004). A two‐way *t*‐test analysis of variance was used to determine statistical differences between the stability of the individual domains. Statistical significance was determined by a value of *p* < 0.01 when comparing four trials for each domain.

### 5.4 Circular dichroism

Circular Dichroism (CD) spectra of 100 μg/ml samples of I82‐I83 were measured at 20°C with a JASCO CD Spectrophotometer, model J‐1500 (JASCO International Co., Tokyo, Japan). Far‐UV measurements in the 190 to 250‐nm range were made every 0.1 nm at a rate of 100 nm/min using a quartz cuvette with a 0.1 cm path length. Temperature was regulated using a Koolance water circulator. Spectra are the average of four scans and were background subtracted before deconvolution. The signal (mdeg) was converted to molar ellipticity (θ) using Equation (5):

(5) $$\theta =\frac{\text{mdeg}\cdot M}{C\cdot L\cdot 10}$$

in which *M* is the molecular weight, *L* is the path length of the cell in centimeters, and *C* is the concentration of the protein in g/L. Secondary structure analysis of CD spectra were completed using K2D3 (Louis‐Jeune et al., 2012).

### 5.5 NMR spectroscopy

Sample preparation and NMR data collection for the I82‐I83 tandem was done as previously for the individual domains on their own (Kelly, Pace, et al., 2021). Default conditions for NMR experiments with the tandem were: concentration: 0.5 mM; buffer: 100 mM NaCl, 20 mM HEPES pH 7.0, 2 mM DTT, 0.02% NaN3; temperature: 298 K; magnetic field: 800 MHz using a Bruker Neo spectrometer equipped with a TCI cryoprobe. Data collection and processing were done with Topspin 4.3.0 while analysis of spectra and assignment was done with CCPNMR AnalysisAssign 3.2 (Skinner et al., 2016). The ^1^H‐^15^N‐HSQC spectrum (default Bruker pulse sequence: hsqcwg) of the tandem domain was assigned by superposing it on the spectra of the individual domains I82 & I83 separately (Figure 3). For most residues, peak positions in the tandem are virtually identical to those in the individual domains, so that those could be assigned without further assessment. To assign those amino acids with larger chemical shift perturbations (CSP), an initial search was performed in the vicinity of the peak of the corresponding amino acid in the individual domains. The nearest unassigned peaks in the tandem spectrum were considered as candidates for assignment. To distinguish them, a ^15^N resolved 3D NOESY‐HSQC experiment (default Bruker pulse sequence: noesyhsqcf3gpwg3d) was conducted. NOESY peak patterns of the candidate peaks in the 2D HSQC spectrum of the tandem were compared to (a) the NOESY peak patterns of the corresponding peak in the individual domain and (b) the NOESY peak pattern of the preceding/following residue in the ^15^N 3D NOESY‐HSQC spectrum of the tandem. Only once both criteria were met did we consider the peak as safely assigned. In this way all non‐proline amino acids could be assigned in the tandem.

With the assigned peaks in the ^1^H‐^15^N HSQC of the tandem it was possible to calculate backbone CSPs for the comparison of the tandem to the individual domains for all amino acids by comparing peak positions of the same amino acid in the individual domains vs. the tandem (Figure 3). The CSP value was calculated as the weighted absolute differences of the peak positions in both ^1^H and ^15^N dimensions:

$$\mathrm{CSP}=\sqrt{0.2*\delta {\left(Ν\right)}^{2}+\delta {\left(Η\right)}^{2}}$$

With the full assignment of the backbone ^1^H & ^15^N resonances we performed a partial side chain ^1^H assignment using the ^15^N resolved 3D NOESY experiment. 2D ^1^H‐^1^H NOESY strips for each residue were taken from the corresponding experiments for the individual domains as well as for the tandem and superposed or placed side by side (see Figure 4). The NOEs of the amide protons of G21 and V68 to F121 Hepsilon were assigned based on the following considerations: (1) In isolated I82 these NOEs are absent. (2) The new cross peaks in the 15 N NOESY strips of G21 and V68 have a chemical shift that can really only come from an aromatic ring proton. (3) There are no aromatic rings in I82 in the vicinity of G21 & V68 that could move close enough to generate a NOESY cross peak in the tandem. (4) The only available ring protons in the vicinity of G21 and V68 are across the interface from F121 in domain I83. (5) A likely involvement of F121 in the interface is suggested by the chemical shift changes of the side chain protons of E20.

For all amino acids with a backbone CSP around or below 1σ it was assumed that side chain peak positions in the tandem were essentially identical to those in the individual domains. Where this was confirmed by comparison of the 3D NOESY spectra, the corresponding peaks were assigned as in the individual domains. For residues in the interface, that is, those with backbone CSPs well above 1σ, side chain proton peaks in the 3D NOESY were examined more closely. The starting assumption was that even for the interface the ^1^H chemical shifts should not have changed too much. This is based on the general observation that side chain proton chemical shifts are more determined by the covalent structure of the amino acid than conformation or contacts (Wishart & Sykes, 1994). Therefore, where peaks in the tandem 3D NOESY experiment could be observed at chemical shifts close to those in the individual domains it was assumed that these would be the same resonances, and they were assigned correspondingly. A good example for this approach is shown in Figure 4 for the sidechain resonances of E20. In the spectrum of I82, the two β‐protons have virtually identical chemical shifts leading to an elongated peak at ~2 ppm. In the tandem spectrum, we see two clearly distinct peaks for the β‐protons at 2.0 and 2.2 ppm while the peak for the γ‐protons remains in its original position. It is of course also conceivable that more complex permutations of β‐ and γ‐proton positions lead to the observed spectrum. However, the chosen approach makes the least assumptions and is thus the safest. It is sufficient to be certain that there are changes in some of the sidechain protons to test the validity of the model. Similarly, the assignment of the Hε resonance of F121 is based on the comparison of the tandem NOESY spectrum to those of the individual domains. The NOESY strips of F121 and the following D122 show crosspeaks very close to the positions of the Hδ and Hε protons in I83 alone. Consequently, these were assigned also in the tandem domain.

^15^N relaxation data were collected using standard Bruker pulse sequences (R1: hsqct1etf3gptcwg3d, R2: hsqct2etf3gptcwg3d, heteronuclear NOE: hsqcnoef3gpsi). Relaxation data points were collected at times of 31, 62, 154, 308, 462, 616, 770, 924, 1078, 1232, 1386, 1540 ms for R1 and 12.9, 25.8, 38.7, 51.6, 64.6, 77.5, 90.4, 103.3, 1116.2, 142.0, 167.8, 193.7 ms for R2. For the heteronuclear NOE two individual HSQC spectra were recorded in interleaved mode where ^1^H saturation via a 3 s pulse train was alternated between off and on. The heteronuclear NOE was calculated as the ratio of the peak intensity of the saturated peak divided by the intensity of the non‐saturated peak. Raw relaxation data were then analyzed using the Lipari–Szabo approach first to calculate the overall rotation tumbling rate τ_c_ using the R1/R2 ratios for residues with heteronuclear values >0.6. Given the anisotropy of the molecule, the isotropic tumbling model could not be used here. Instead, it was necessary to use an axially symmetric model with an isotropic τ_c_ and a ratio of parallel to perpendicular rotation (D||/D⊥) implemented in the software R1R2diffusion (Mandel et al., 1995; Palmer III et al., 1991). The valueσ of τ_c_ and D||/D⊥ were then used in a residue‐by‐residue analysis of local dynamics using the software modelfree (Palmer, 2004). In essence, parameters describing the amplitude and time scale of local motions are extracted from the raw relaxation parameters. The most important of those are the order parameter S^2^ which informs about the amplitude of local motions on the ps time scale with a maximum value of 1.0 for absolute rigidity and 0.0 for absolute freedom; the exchange line broadening R_ex_ indicates the presence of motions on the ms time scale and the order parameter S^2^_i_ which is identical to the order parameter but informs on motions on the low ns time scale.

Interaction of the tandem with calcium was measured by the comparison of ^1^H‐^15^N HSQC spectra (see above) recorded under standard conditions in the absence and presence of a saturating concentration of CaCl_2_ (10 mM). CSPs were calculated as described above for peak differences between the two spectra for all assigned amino acids.

To obtain ^1^H‐^15^N residual dipolar couplings (RDC) for structure calculation we recorded in‐phase/antiphase (IPAP) spectra using a ^15^N labeled sample of the tandem construct under standard conditions in the absence and presence of 10 mg/mL of Pf1 phage (Asla Biotech) using a standard Bruker pulse sequence (hsqcf3gpiaphwg.2). ^1^H‐^15^N couplings were extracted for a total of 172 residues in both experiments from the chemical shift difference of the doublet peak in the ^15^N frequency axis. Subsequently, the differences of these couplings in the presence and absence of alignment medium (Pf1 phage) were calculated to yield the RDCs.

### 5.6 Structure calculation & analysis

The structure of the tandem was calculated based on the structures of the two individual domains I82 and I83 (PDB entries 9IBI/9IBK for I82 and 6YJ0 for I83) using the HADDOCK 2.4 webserver (Honorato et al., 2021; Honorato et al., 2024). Default parameters were used in most cases with only a few adapted (structures for docking: 6000; trials for rigid body minimization: 10; structures for refinement: 500; final refinement: 500; structures to analyze: 500; SANI constants: 1.5–2.0–3.0). Experimental constraints used for the structure calculation were contact residues (known in HADDOCK as active residues), RDCs and short, NOESY‐derived ^1^H‐^1^H distances. In addition, distance restraints to maintain the core structure of the two domains as well as the distance of the last residue of I82 to the first residue of I83 were added. As active residues we selected amino acids with CSPs >2σ (Figure 5) except for residues 91–96 as CSPs here are more likely to be caused by changes in covalent bonds rather than noncovalent contact. For I82 these are: 18, 19, 20, 21, 22, 68, 69, 70, 71, 72, 74. For I83 these are: 34, 35, 36, 37, 38, 85, 86, 88, 89, 90 (full‐length numbering: 120, 121, 122, 123, 124, 171, 172, 174, 175, 176). To allow for the otherwise rigid domain structures to adapt in the contact interface all residues in the interface were defined as flexible, including the linker sequence between I82 & I83 and the N‐ and C‐termini of the tandem. For I82 these are: 18, 19, 20, 21, 22, 67, 68, 69, 70, 71, 72, 73, 74, 90, 91, 92, 93. For I83 these are: 8, 9, 10, 34, 35, 36, 37, 38, 85, 86, 87, 88, 89, 90 (full‐length numbering: 94, 95, 96, 120, 121, 122, 123, 124, 171, 172, 173, 174, 175, 176). A total of 172 RDC values (see above for the experimental measurement) were used. The alignment tensors were calculated for the individual domains using the individual structures separately and only the corresponding RDC values using PALES (Zweckstetter, 2008). Their averages were used to calculate the alignment tensor for the complete protein as input for HADDOCK (I82: Da = 5.55; Rh = 0.27. I83: Da = 7.26; Rh = 0.65. Averages: Da = 6.4; Rh = 0.45). The 3D ^15^N resolved NOESY‐HSQC experiment allowed the identification of two ^1^H‐^1^H cross peaks from the comparison of equivalent 2D strips of the 3D NOESY experiments of the tandem and the individual domains: Hε of F121 to HN of G21 as well as V68 (Figure 4). These two distances were calibrated at 5.0 Å. Additional distance constraints were used for all CA‐CA distances within 6 Å within each domain to maintain the structure of the domains during the calculation (early attempts at docking without such constraints had shown distortions in the domain structures). Additionally, a distance constraint of 1.5 Å between the C‐terminal carboxyl carbon of I82 and the N‐terminal amide nitrogen of I83 was introduced to ensure the correct positioning of the domains to introduce a covalent link after docking. Prior to docking the flexible termini were removed from the experimental structure of each domain, leaving residues F5‐A93 for I82 and E8‐C105 (full‐length numbering: E94‐C191) for I83. The residues in each domain were subsequently renumbered to match the numbering of the full‐length construct. The best cluster of each calculation was selected based on the number of structures represented (the more the better), RMSD (the lower the better), best agreement with experimental data and overall quality (Figure S2). All structure manipulation, analysis and preparation of structure figures were done with Yasara (Krieger & Vriend, 2014). Individual domains in the tandem structures from the two selected clusters were combined into a single protein using a home‐written macro in Yasara (Yasara has its own macro language called Yanaconda). Its syntax is described in the Yasara manual (Krieger & Vriend, 2015). In short, atoms surplus to requirements at the C‐terminus of I82 and N‐terminus of I83 were removed, a new bond was added and a short, simulated annealing energy minimization was performed to optimize the geometry and reduce clashes around the new bond. All further analysis of solution and NMR structures were carried out with built‐in functions of Yasara such as simultaneous superposition (*SubMultiObj* which uses the Theseus maximum likelihood algorithm; Theobald & Wuttke, 2006) and calculation of solvent‐exposed surfaces (*ConSurfRes*).

The angle between the principal axes of I82 and I83 in the tandem was calculated with the Yasara command *GroupAngle* using the backbone atoms of residues 8–18 23–31 37–41 52–66 73–92 in I82 and 96–107,111–120,124–131,140–156,163–171,175–187 covering essentially the β‐strands. The same selection was used with the command *GroupDihedral* to calculate the angle formed by the planes defined by the β‐sheets in each domain, that is, the twist of the domains around the common principal axis.

## AUTHOR CONTRIBUTIONS

**Colleen M. Kelly:** Conceptualization; investigation; writing – original draft; methodology; writing – review and editing; formal analysis; data curation. **Janette Jerusal:** Investigation. **Mark Pfuhl:** Investigation; writing – original draft; methodology; writing – review and editing; formal analysis; data curation; resources. **Matthew J. Gage:** Conceptualization; investigation; writing – original draft; methodology; writing – review and editing; project administration; supervision; resources.

## Supporting information


**FIGURE S1.** Differences in the fluorescence spectra indicate change in local environment of tryptophan at 50 μM Ca^2+^ (pCa 4.3). (a) The CoM of the emission curve for the native tandem construct at 50 μM is lower than it is in the absence of calcium, indicating that one or more of the tryptophan are in a more hydrophobic environment after a 1‐h incubation in the presence of calcium. The unfolded state yielded a similar CoM in both environments. (b) A decrease in the CD signal for the tandem I82‐I83 in the presence of 50 μM Ca^2+^ demonstrates that the structure undergoes a conformational change under these conditions with an increase in alpha helical character. The solid black curve is the spectrum for I82‐I83 in the absence of Ca^2+^ and the gray curve is the spectrum for the I82‐I83 in the presence of 50 μM Ca^2+^.
**FIGURE S2.** Representative raw data from stability experiments. Fluorescence is shown on the Y‐axis and wavelength is shown on the X‐axis.
**FIGURE S3.** Representative plot showing Center of Mass verse urea used to determine baselines for linear extrapolation fitting. The upper and lower lines are shown to highlight which points were used to establish the baselines.
**FIGURE S4.** Plot of experimental RDC values of 182‐183 in 10 mg/ml of Pf1 phase against the values predicted by PALES for the best model built by HADDOCK.
**FIGURE S5.** (a) Reconstruction of the crystal lattice of the 181‐182‐183 tandem construct of human titin (PDB entry: 7AHS). The 3D lattice was reconstructed with ChimeraX using the space group information P 21 21 21 and then analyzed in Yasara. A selection of molecules are shown in an arbitrary colors. Despite the overlap of multiple molecules the main orientations along the vertical and the horizontal are evident. (b) Detailed view of four molecules shown in different colors to illustrate some of the crystal contacts (middle). On the left and right different contacts are shown in detail with the covered solvent accessible surface shown next.

## Data Availability

The NMR assignments for the I82‐I83 structure have been uploaded to the PDB (Entry Number: 9RHK). All other data and the clones used in this project are available upon request.
