# Supplementary material for: Calcium‐dependent cooperativity and stability of Titin's tandem I82‐I83 domains
Source: Protein Sci. 2025 Nov 12;34(12):e70378. doi: 10.1002/pro.70378 (PMC12611880; doi:10.1002/pro.70378)

I82-I83 Supplemental Data:

Kelly et al


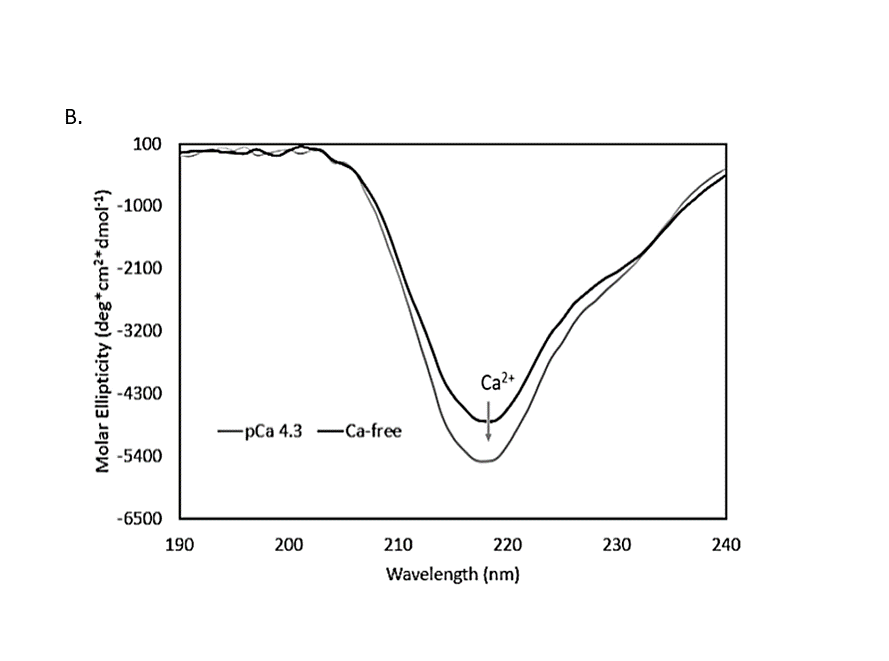

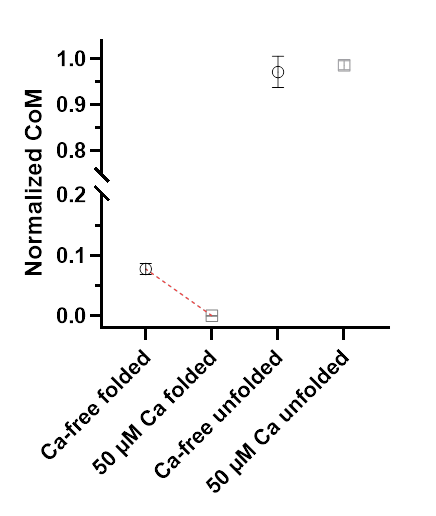
Figure S1

B.

A.

**Figure S1. Differences in the fluorescence spectra indicate change in local environment of tryptophan at 50 µM Ca^2+^ (pCa 4.3).** (A) The CoM of the emission curve for the native tandem construct at **50 µM** is lower than it is in the absence of calcium, indicating that one or more of the tryptophan are in a more hydrophobic environment after a 1-hr incubation in the presence of calcium. The unfolded state yielded a similar CoM in both environments. (B) A decrease in the CD signal for the tandem I82-I83 in the presence of **50 µM Ca^2+^** demonstrates that the structure undergoes a conformational change under these conditions with an increase in alpha helical character. **The solid black curve is the spectrum for I82-I83 in the absence of Ca^2+^ and the grey curve is the spectrum for the I82-I83 in the presence of 50 µM Ca^2+^.**

**Muscle physiologists report calcium concentrations using pCa, which is calculated as pCa = -log [Ca^2+^]. Using this formula, pCa 4.3 = 50 µM Ca^2+^.**

Kelly et al

Figure S2

**Figure S2. Representative raw data from stability experiments. Fluorescence is shown on the Y-axis and wavelength is shown on the X-axis.**

Kelly et al

Figure S3

**Figure S3. Representative plot showing Center of Mass verse urea used to determine baselines for linear extrapolation fitting. The upper and lower lines are shown to highlight which points were used to establish the baselines.**

Kelly et al

Figure S4


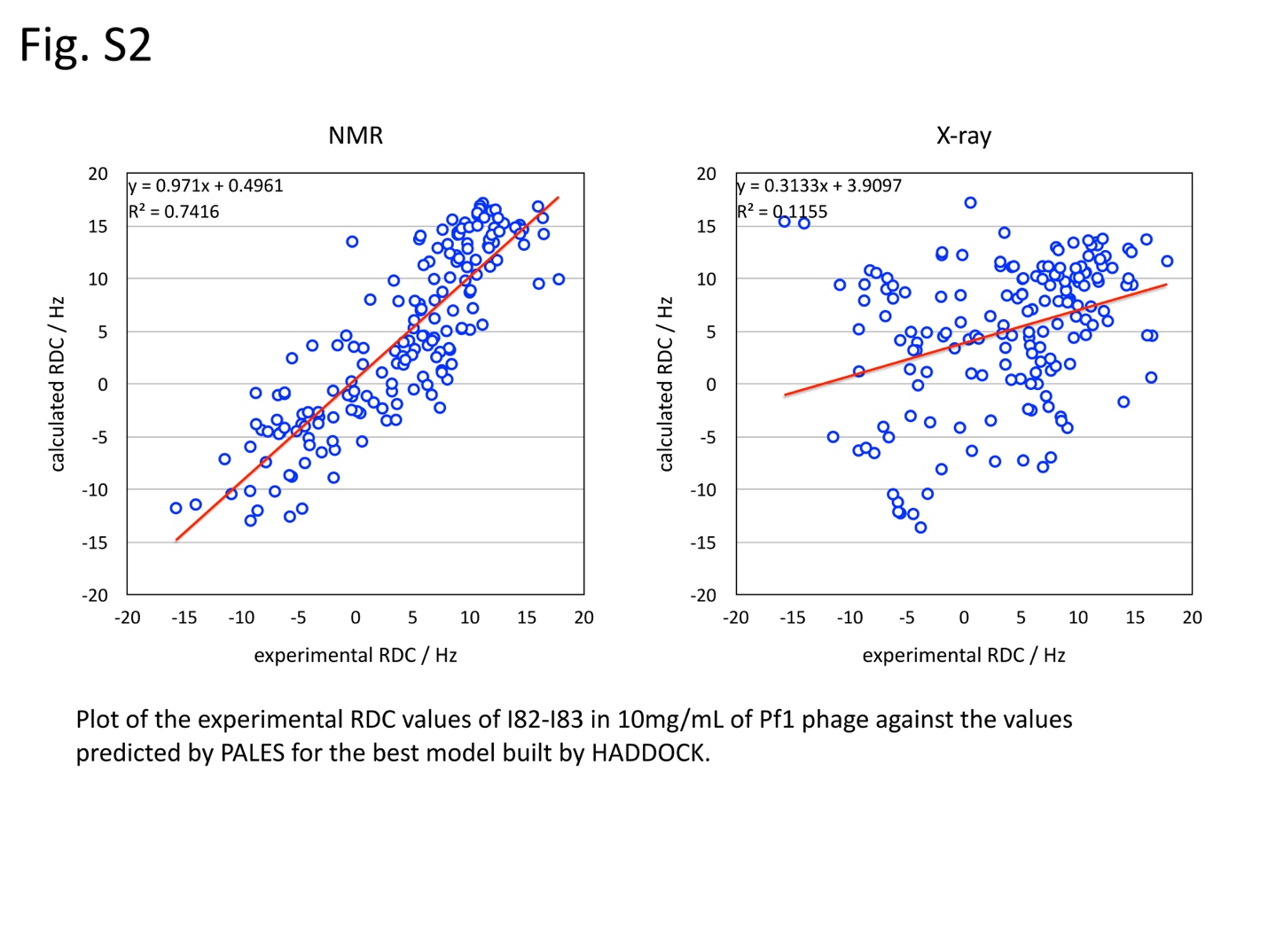


Kelly et al

Figure S5A


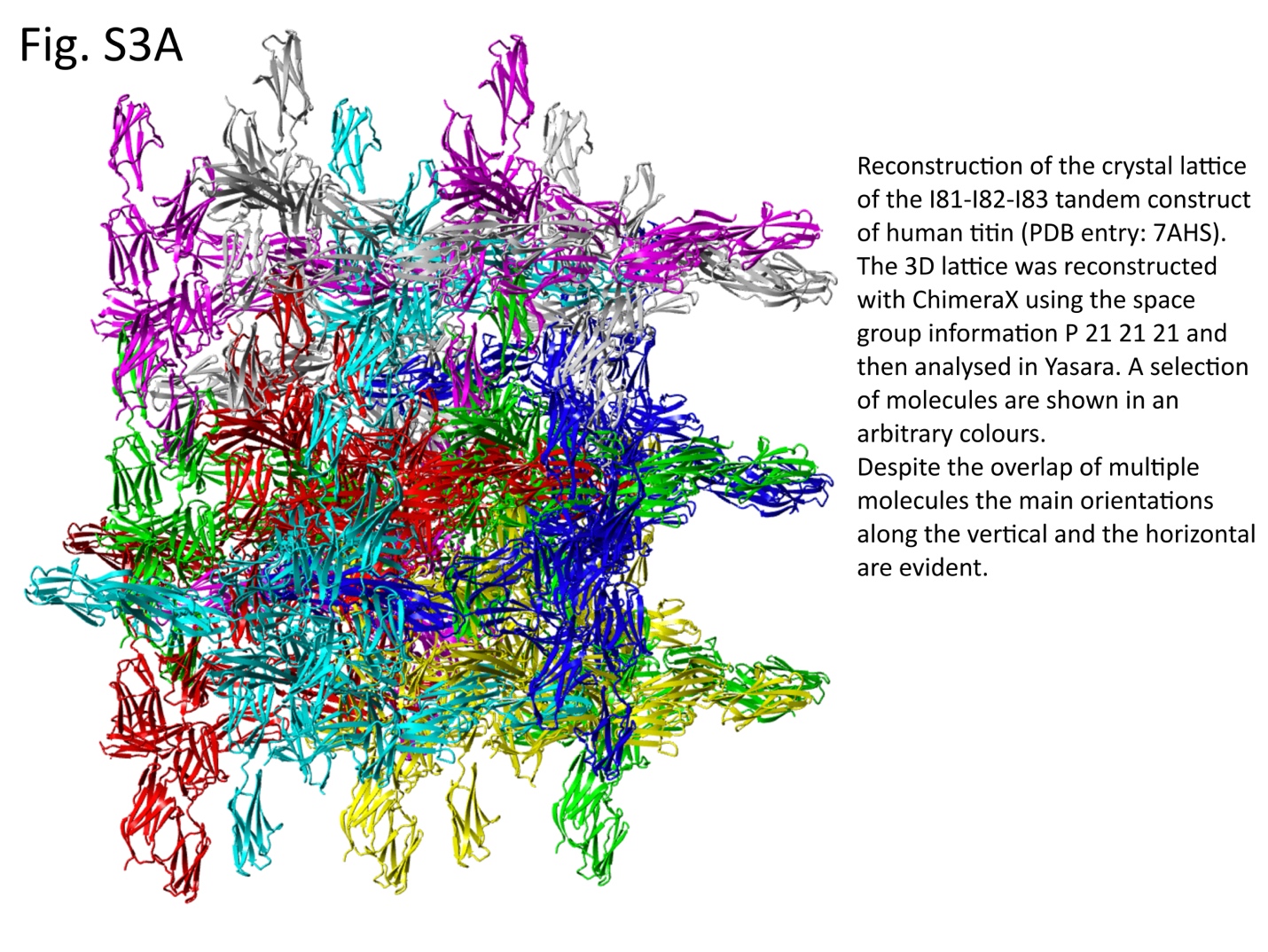


Kelly et al

Figure S5B


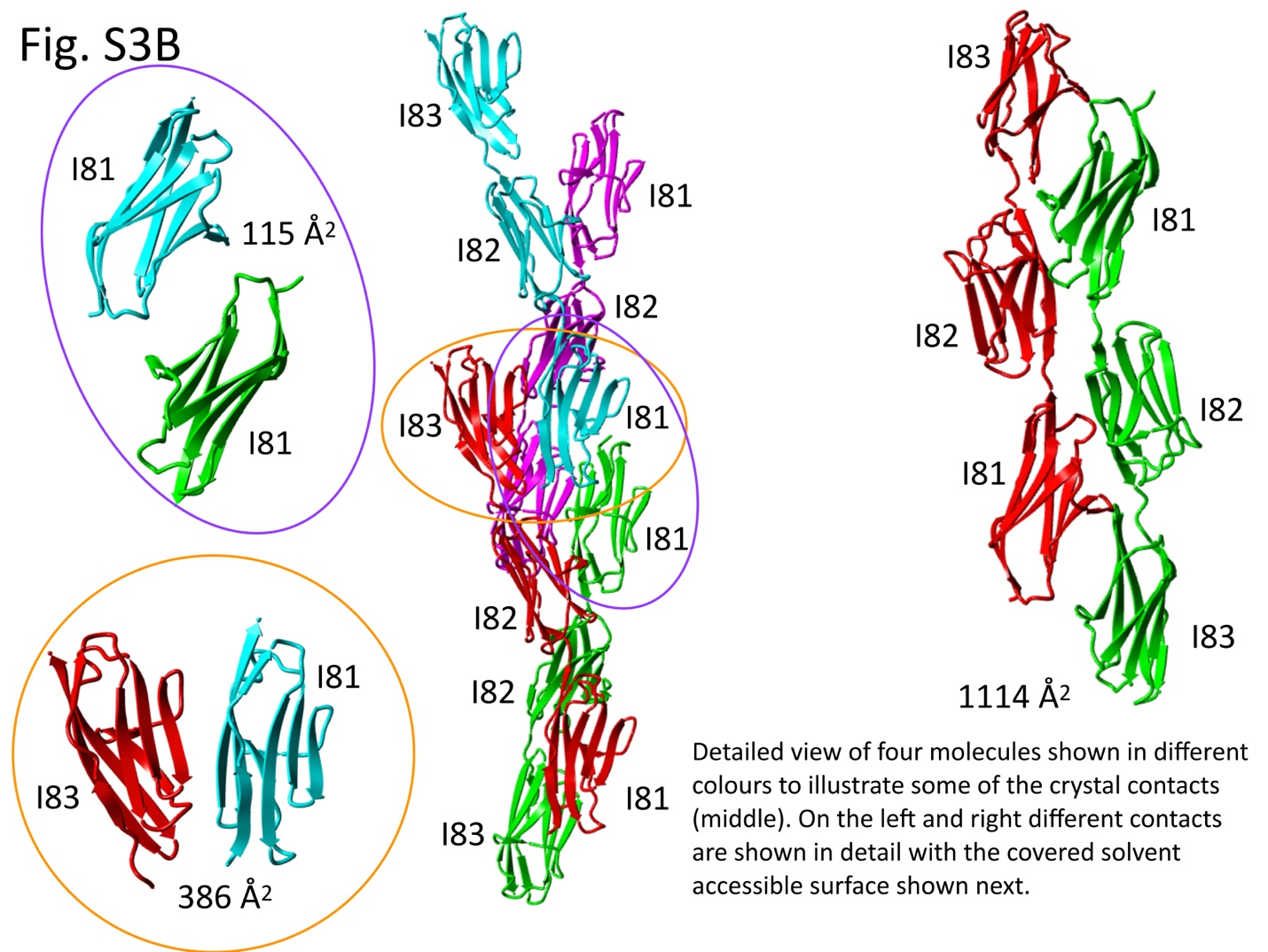

Supplement: Supplementary file 1 — FIGURE S1. Differences in the fluorescence spectra indicate change in local environment of tryptophan at 50 μM Ca2+ (pCa 4.3). (a) The CoM of the emission curve for the native tandem construct at 50 μM is lower than it is in the absence of calcium, indicating that one or more of the tryptophan are in a more hydrophobic environment after a 1‐h incubation in the presence of calcium. The unfolded state yielded a similar CoM in both environments. (b) A decrease in the CD signal for the tandem I82‐I83 in the presence of 50 μM Ca2+ demonstrates that the structure undergoes a conformational change under these conditions with an increase in alpha helical character. The solid black curve is the spectrum for I82‐I83 in the absence of Ca2+ and the gray curve is the spectrum for the I82‐I83 in the presence of 50 μM Ca2+. FIGURE S2. Representative raw data from stability experiments. Fluorescence is shown on the Y‐axis and wavelength is shown on the X‐axis. FIGURE S3. Representative plot showing Center of Mass verse urea used to determine baselines for linear extrapolation fitting. The upper and lower lines are shown to highlight which points were used to establish the baselines. FIGURE S4. Plot of experimental RDC values of 182‐183 in 10 mg/ml of Pf1 phase against the values predicted by PALES for the best model built by HADDOCK. FIGURE S5. (a) Reconstruction of the crystal lattice of the 181‐182‐183 tandem construct of human titin (PDB entry: 7AHS). The 3D lattice was reconstructed with ChimeraX using the space group information P 21 21 21 and then analyzed in Yasara. A selection of molecules are shown in an arbitrary colors. Despite the overlap of multiple molecules the main orientations along the vertical and the horizontal are evident. (b) Detailed view of four molecules shown in different colors to illustrate some of the crystal contacts (middle). On the left and right different contacts are shown in detail with the covered solvent accessible surface shown next. [file PRO-34-e70378-s001.docx]
